# Ab initio electron-two-phonon scattering in GaAs from next-to-leading order perturbation theory

**DOI:** 10.1038/s41467-020-15339-0

**Published:** 2020-03-30

**Authors:** Nien-En Lee, Jin-Jian Zhou, Hsiao-Yi Chen, Marco Bernardi

**Affiliations:** 1 Department of Applied Physics and Materials Science, California Institute of Technology, Pasadena, CA 91125 USA; 2 Department of Physics, California Institute of Technology, Pasadena, CA 91125 USA

**Keywords:** Computational methods, Electronic structure, Electronic properties and materials, Semiconductors

## Abstract

Electron-phonon (*e*–ph) interactions are usually treated in the lowest order of perturbation theory. Here we derive next-to-leading order *e*–ph interactions, and compute from first principles the associated electron-two-phonon (2ph) scattering rates. The derivations involve Matsubara sums of two-loop Feynman diagrams, and the numerical calculations are challenging as they involve Brillouin zone integrals over two crystal momenta and depend critically on the intermediate state lifetimes. Using Monte Carlo integration together with a self-consistent update of the intermediate state lifetimes, we compute and converge the 2ph scattering rates, and analyze their energy and temperature dependence. We apply our method to GaAs, a weakly polar semiconductor with dominant optical-mode long-range *e*–ph interactions. We find that the 2ph scattering rates are as large as nearly half the value of the one-phonon rates, and that including the 2ph processes is necessary to accurately predict the electron mobility in GaAs from first principles.

## Abstract (web summary)

Electron-phonon scattering plays a decisive role in electron transport and is taken into account in ab initio calculations by leading-order perturbations involving scattering events with one phonon. Here, the authors show that higher-order effects are comparable in magnitude to the leading order in polar semiconductors.

## Introduction

Electron–phonon (*e*–ph) interactions are essential to understanding electrical transport, nonequilibrium dynamics and superconductivity. Using density functional theory (DFT) and related methods, it has become possible to compute *e*–ph interactions from first principles, and use them to predict the carrier scattering rates and mobilities, both in simple and in complex materials with up to tens of atoms in the unit cell^1–9^. In the typical workflow, one takes into account only the leading-order *e*–ph scattering processes, which involve scattering of the carriers with one phonon. Nearly all ab initio work to date has relied on such leading-order perturbation theory, tacitly neglecting higher-order *e*–ph processes.

Yet many compounds, including polar semiconductors, oxides and organic crystals, exhibit *e*–ph interactions that cannot be treated with lowest-order perturbation theory. Polar materials are a paradigmatic case in which *e*–ph interactions due to polar optical modes—in particular, the longitudinal optical (LO) mode—are long-range^10,11^, which can lead to higher-order *e*–ph scattering and polaron formation. In weakly polar materials, such as III-V and II-VI semiconductors and high-mobility oxides, one expects that perturbation theory is still valid, but that higher-order *e*–ph processes are significant and need to be included.

Studies of second- and even third-order *e*–ph processes in semiconductors exist^12–17^, but they are limited to simplified models, restricted to the conduction band minimum, or only valid at zero temperature, and are therefore inadequate for quantitative predictions. In first-principles *e*–ph and charge transport calculations, next-to-leading-order effects have been typically assumed to be negligible, even though their contributions have been estimated to be important using simplified models^13–15,18,19^. In materials with intermediate or strong *e*–ph coupling, such as oxides with low room-temperature mobility and ionic compounds (e.g., alkali halides), the *e*–ph interactions can lead to regimes beyond the reach of perturbation theory, including the formation of polarons^20^. This coupling regime has been investigated with diagram-resummation techniques such as the cumulant method, both analytically^21^ and more recently ab initio^22–24^.

Higher-order processes are generally important in quantum field theories of condensed matter. An example are light–matter interactions, where phonon-assisted^25^ and two-photon^26,27^ absorption have been studied extensively. Even calculations in quantum electrodynamics beyond the leading order can provide important lessons—for example, higher-order corrections are essential to accurately predict large-angle Bhabha scattering in electron-positron collisions^28^, and calculations up to the tenth order have been carried out to compute the magnetic moment of the electron^29^.

**Fig. 1:**
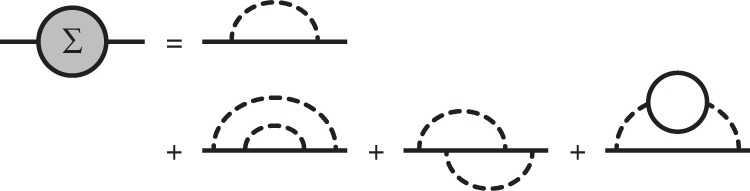
Next-to-leading-order self-energy diagrams. Diagrams for the *e*-ph self-energy up to ${\mathcal{O}}$(*g*^4^), where *g* is the *e*–ph coupling constant. The first two diagrams in the second row contribute to the two-phonon scattering processes.

However, it is a daunting task to systematically go beyond the leading order due to the rapid increase in the number of Feynman diagrams and their computational complexity. For *e*–ph interactions, the next-to-leading order involves electron scattering events with two phonons, which requires computationally challenging Brillouin zone integrals over two crystal momenta. This computational complexity has hampered next-to-leading-order *e*–ph calculations for decades^12^, and the relevant next-to-leading-order diagrams (see Fig. 1) have not yet been computed numerically from first principles.

Here we formulate and compute from first principles next-to-leading-order *e*–ph interactions, focusing on electron scattering processes involving two phonons (hereafter denoted as 2ph processes). We compute and analyze their contributions to the *e*–ph scattering rates, using as an ideal case study a weakly polar semiconductor, GaAs. We find that the 2ph scattering rates are surprisingly large, and comparable in magnitude to the lowest-order rates due to one-phonon (1ph) processes. Our analysis shows that the relative importance of the 2ph contributions is nearly temperature independent at 200–400 K, and rationalizes the peculiar energy dependence of the 2ph processes. The results are sensitive to the lifetimes of the intermediate states, which need to be included to avoid divergences due to resonance effects; we develop a self-consistent scheme to overcome this challenge. The 2ph processes are also shown to play an important role in accurately predicting the electron mobility in GaAs. We formulate and iteratively solve a linearized Boltzmann transport equation (BTE) that includes the 2ph processes, showing that this level of theory can correct the discrepancy with experiment of the mobility predicted with the BTE including only 1ph processes. Our work proposes an approach for systematically improving the accuracy of ab initio *e*–ph calculations beyond the leading order. While this method is broadly applicable, it is particularly well-suited for weakly polar (III-V and II-VI) semiconductors and high-mobility oxides, in which the weak polaron effects can be treated perturbatively.

## Results

### Two-phonon processes and their scattering rates

We use the Matsubara frequency sum technique^30^ to derive the electron self-energy due to *e*–ph interactions up to ${\mathcal{O}}$(*g*^4^) (see Fig. 1), where *g* is the *e*–ph coupling constant; our treatment focuses on computing the imaginary part of the self-energy and the related 2ph scattering rates. The derivations are lengthy and tedious, and are given in detail in the Supplementary Information.

**Fig. 2:**
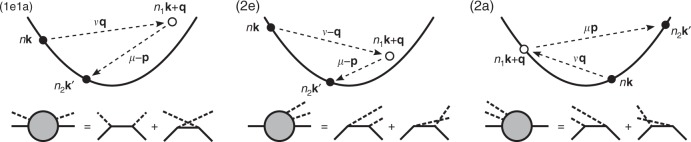
Electron-two-phonon scattering processes. Two-phonon scattering processes considered in this work, including one-phonon absorption plus one-phonon emission (left panel, labelled 1e1a), two-phonon emission (middle panel, labelled 2e) and two-phonon absorption (right panel, labelled 2a). Each of these three processes comprises two interfering scattering channels, only one of which is shown in the band structure schematics. The lower panels show the corresponding Feynman diagrams with two external phonon lines. Note that the intermediate state does not need to be on-shell; of the three processes shown here, only the 2a is on shell.

All 2ph processes considered in this work consist of two consecutive 1ph scattering events^12^, as we show in Fig. 2. They include processes in which the electron absorbs one phonon and emits another phonon, or vice versa (both processes are denoted as 1e1a), and processes in which the electron emits or absorbs two phonons (denoted as 2e and 2a, respectively). The intermediate electronic state accessed after the first scattering event is associated with a virtual electron that cannot be observed and whose energy can take any value. Processes are defined as on shell when the intermediate electron energy equals a band electron energy, and off shell when it does not (see Fig. 2). As we discuss below, on-shell processes lead to divergent integrals, which are regularized by including the intermediate electron lifetime.

The key result of our analysis is the scattering rate due to the 2ph processes, ${\Gamma }_{n{\bf{k}}}^{(2{\rm{ph}})}$, for an electronic state with band index *n* and crystal momentum **k**, which can be expressed as

1 $${\Gamma }_{n{\bf{k}}}^{(2{\rm{ph}})}=\frac{2\pi }{\hslash }\frac{1}{{N}_{\Omega }^{2}}\ \mathop{\sum }_{{n}_{2}}\ \mathop{\sum }_{\nu {\bf{q}}}\ \mathop{\sum }_{\mu {\bf{p}}}\left[\ {\widetilde{\Gamma }}^{(1{\rm{e}}1{\rm{a}})}+{\widetilde{\Gamma }}^{(2{\rm{e}})}+{\widetilde{\Gamma }}^{(2{\rm{a}})}\right],$$

where the process-resolved 2ph scattering rates ${\widetilde{\Gamma }}^{({i})}$ (*i* = 1e1a, 2e or 2a) depend on the two-phonon momenta **q** and **p** and their respective mode indexes *ν* and *μ*, and *n*_2_ is the band index of the final electronic state, whose momentum is fixed to ${{\bf{k}}}^{\prime}\equiv {\bf{k}}+{\bf{q}}+{\bf{p}}$ by momentum conservation; to correctly normalize the sum, we divide it by ${N}_{\Omega }^{2}$, the number of points sampled in the (**q**, **p**) space. The process-resolved 2ph scattering rates are defined as

2 $${\widetilde{\Gamma }}^{({{i}})}={\gamma }^{({{i}})}\ \delta ({\varepsilon }_{n{\bf{k}}}-{\varepsilon }_{{n}_{2}{{\bf{k}}}^{\prime}}-{\alpha }_{{\bf{p}}}^{({{i}})}{\omega }_{\mu {\bf{p}}}-{\alpha }_{{\bf{q}}}^{({{i}})}{\omega }_{\nu {\bf{q}}}),$$

where *ε* are electron energies relative to the chemical potential and *ω* are phonon energies; the delta function imposes energy conservation and the constants *α* for each process are defined as

$$\, {\alpha }_{{\bf{p}}}^{(1{\rm{e}}1{\rm{a}})}=\ \ 1,\ \ {\alpha }_{{\bf{p}}}^{(2{\rm{e}})}=1,\ \ {\alpha }_{{\bf{p}}}^{(2{\rm{a}})}=-1,\\ \, {\alpha }_{{\bf{q}}}^{(1{\rm{e}}1{\rm{a}})}=-1,\ \ {\alpha }_{{\bf{q}}}^{(2{\rm{e}})}=1,\ \ {\alpha }_{{\bf{q}}}^{(2{\rm{a}})}=-1.$$

The square amplitudes of the three processes are

3 $${\gamma }^{({{i}})}= 	\ {A}^{({{i}})}\times \\ 	\,\left|\sum _{{n}_{1}}\left(\frac{{g}_{{n}_{1}n\nu }({\bf{k}},{\bf{q}}){g}_{{n}_{2}{n}_{1}\mu }({\bf{k}}+{\bf{q}},{\bf{p}})}{{\varepsilon }_{{n}_{2}{{\bf{k}}}^{\prime}}-{\varepsilon }_{{n}_{1}{\bf{k}} + {\bf{q}}}+{\alpha }_{{\bf{p}}}^{({{i}})}{\omega }_{\mu {\bf{p}}}+{{i}}\, \eta -{\Sigma }_{{n}_{1}{\bf{k}} + {\bf{q}}}}\right.\right.\\ 	\,+{\left.\left.\frac{{g}_{{n}_{1}n\mu }({\bf{k}},{\bf{p}}){g}_{{n}_{2}{n}_{1}\nu }({\bf{k}}+{\bf{p}},{\bf{q}})}{{\varepsilon }_{{n}_{2}{{\bf{k}}}^{\prime}}-{\varepsilon }_{{n}_{1}{\bf{k}}+{\bf{p}}}+{\alpha }_{{\bf{q}}}^{({{i}})}{\omega }_{\nu {\bf{q}}}+{{i}}\, \eta -{\Sigma }_{{n}_{1}{\bf{k}} + {\bf{p}}}}\right)\right|}^{2},$$

where *n*_1_ is the band index, Σ is the self-energy of the intermediate electronic state and *η* is a positive infinitesimal. The prefactors *A*^(*i*)^ contain the thermal occupation numbers of electrons and phonons (denoted by *f* and *N*, respectively) and are defined as:

4 $${A}^{(1{\rm{e}}1{\rm{a}})}= \, {N}_{\nu {\bf{q}}}+{N}_{\nu {\bf{q}}}\, {N}_{\mu {\bf{p}}}+{N}_{\mu {\bf{p}}}\, {f}_{{n}_{2}{{\bf{k}}}^{\prime}}-{N}_{\nu {\bf{q}}}\; {f}_{{n}_{2}{{\bf{k}}}^{\prime}},\\ {A}^{(2{\rm{e}})}= \, \frac{1}{2}\left[(1+{N}_{\nu {\bf{q}}})(1+N_{\mu {\bf{p}}}- f_{{n}_{2}{{\bf{k}}}^{\prime}})-{N}_{\mu {\bf{p}}}\; {f}_{{n}_{2}{{\bf{k}}}^{\prime}}\right],\\ {A}^{(2{\rm{a}})}= \, \frac{1}{2}\left[{N}_{\nu {\bf{q}}}(N_{\mu {\bf{p}}}+ f_{{n}_{2}{{\bf{k}}}^{\prime}})+(1+ N_{\mu {\bf{p}}}){f}_{{n}_{2}{{\bf{k}}}^{\prime}}\right].$$

**Fig. 3:**
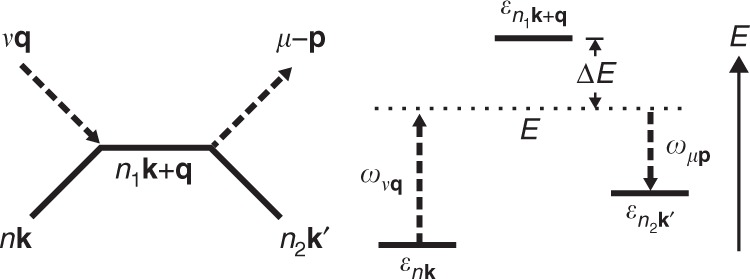
Energetics of two-phonon processes. The example 1e1a process discussed in the text, with its energetics shown on the right.

Let us examine the process amplitudes in Eq. (3). Since all three processes have similar expressions, we focus on a 1e1a process in which the electron absorbs one phonon and then emits another as an example, though our considerations are general. In this process, which is shown schematically in Fig. 3, an electron first absorbs a phonon with energy *ω*_*ν***q**_, transitioning to an intermediate state $\left|{n}_{1}{\bf{k}}+{\bf{q}} \right\rangle$ with energy *E* = *ε*_*n***k**_ + *ω*_*ν***q**_, and then emits a phonon with energy *ω*_*μ***p**_, reaching the final state with energy ${\varepsilon }_{{n}_{2}{{\bf{k}}}^{\prime}}$. Note that the energy *E* of the intermediate (virtual) electron does not need to match its band energy ${\varepsilon }_{{n}_{1}{\bf{k}}+{\bf{q}}}$. The amplitude for this process, from Eq. (3), reads

5 $$\frac{{g}_{{n}_{1}n\nu }({\bf{k}},{\bf{q}}){g}_{{n}_{2}{n}_{1}\mu }({\bf{k}}+{\bf{q}},{\bf{p}})}{{\varepsilon }_{{n}_{2}{{\bf{k}}}^{\prime}}+{\omega }_{\mu {\bf{p}}}-{\varepsilon }_{{n}_{1}{\bf{k}}+{\bf{q}}}-{\Sigma }_{{n}_{1}{\bf{k}}+{\bf{q}}}},$$

where we omitted the *iη* infinitesimal for clarity. This expression can be understood as follows: the two *e*–ph coupling constants are associated with each of the two consecutive 1ph scattering events, and the denominator is due to the propagator of the intermediate electron, which is proportional to $1/(E-{\varepsilon }_{{n}_{1}{\bf{k}}+{\bf{q}}}-{\Sigma }_{{n}_{1}{\bf{k}}+{\bf{q}}})$. The amplitude of the 2ph process is thus inversely proportional to its off-shell extent, $\Delta E=E-{\varepsilon }_{{n}_{1}{\bf{k}}+{\bf{q}}}$, implying that processes with large intermediate off-shell extents Δ*E* are unlikely to occur. In an on-shell 2ph process, *E* is equal (or very close) to the intermediate-state band energy ${\varepsilon }_{{n}_{1}{\bf{k}}+{\bf{q}}}$, resulting in a small denominator Δ*E* ≃ 0 and thus in a large amplitude. The inverse lifetime of the intermediate state, which is proportional to ${\rm{Im}}{\Sigma }_{{n}_{{\rm{1}}}{\bf{k}}+{\bf{q}}}$, prevents the on-shell process amplitude from diverging.

### Numerical calculations and intermediate lifetime

We implement the expressions derived above in our in-house developed code, Perturbo^31^, and carry out first-principles calculations on GaAs (see Methods). In our approach, we sum the 2ph scattering rates in Eq. (1) over all possible pairs of phonons, and thus include both on-shell and off-shell processes on the same footing. The treatment of the intermediate lifetimes in our implementation deserves a detailed discussion. When the intermediate state is on shell, the scattering process results in resonance effects, and as discussed above the intermediate state lifetime is crucial to prevent the 2ph scattering rate from diverging. Here and below, the intermediate state lifetime is defined as the inverse scattering rate of the intermediate state, which is obtained from the imaginary part of its self-energy as $2\ {\rm{Im}}\Sigma /\hslash$.

The 2ph scattering rates depend on the intermediate state self-energy, Σ in the denominator of Eq. (3), whose value needs to be chosen carefully. We neglect its real part, which only corrects the band structure and barely affects the 2ph calculation. The imaginary part of Σ includes in principle scattering effects from all possible sources. In practice, we approximate ${\rm{Im}}\Sigma$ with the total *e*–ph scattering rate, including both the lowest-order (1ph) rates and the 2ph rates, using $\left|{\rm{Im}}\Sigma \right|=\hslash /2[{\Gamma }^{(1{\rm{ph}})}+{\Gamma }^{(2{\rm{ph}})}]$, where Γ^(1ph)^ is the usual 1ph scattering rate^3^. This approach makes Eq. (1) a self-consistent problem. We iterate Eq. (1) until the 2ph scattering rate equals the 2ph contribution to the intermediate state lifetime. In each iteration *m*, the lifetime is due to the sum of the lowest order plus the 2ph scattering rates at the previous iteration, namely, $\left|{\rm{Im}}\Sigma (m)\right|=\hslash /2\ [{\Gamma }^{(1{\rm{ph}})}+{\Gamma }^{(2{\rm{ph}})}(m-1)]$. The initial Γ^(2ph)^ is set to zero, and the convergence process is performed separately at each temperature.

### Analysis of the two-phonon scattering rates

**Fig. 4:**
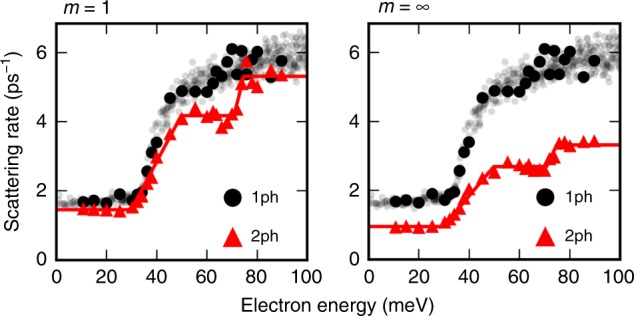
Calculated two-phonon scattering rates. Calculated 2ph scattering rates, ${\Gamma }_{n{\bf{k}}}^{(2{\rm{ph}})}$ in Eq. (1), for electrons in GaAs at 300 K. The zero of the energy axis is the conduction band minimum. The left panel shows the first iteration, and the right panel the final result after converging the intermediate lifetime update procedure. The lowest-order (1ph) *e*–ph scattering rates are also given for comparison.

Figure 4 shows the first iteration and the converged result for the 2ph scattering rates in GaAs at 300 K, for states near the bottom of the conduction band (see Methods). In this energy range, the *e*–ph interactions in GaAs are dominated by the long-range Fröhlich interaction due to the LO mode^3^, with nearly negligible contributions from all other phonon modes. The converged 2ph scattering rates are surprisingly large—they are smaller than the leading-order 1ph rate, thus justifying the perturbative approach, but they are nearly half the value of the 1ph rates at all energies. We find that the rainbow diagram (the first diagram in the second row of Fig. 1) provides the dominant contribution to the 2ph scattering rate, while the contribution from the vertex-correction diagram (the second diagram in the second row of Fig. 1) is negligible, in agreement with Migdal’s theorem. Therefore, lowest-order perturbation theory can capture only part of the dynamical processes due to the long-range *e*–ph interactions with the LO mode in GaAs. Though the results shown in Fig. 4 include only the dominant *e*–ph interaction with the LO mode, we have verified that including all phonon modes gives nearly unchanged 2ph scattering rates in GaAs (see Supplementary Fig. 1).

**Fig. 5:**
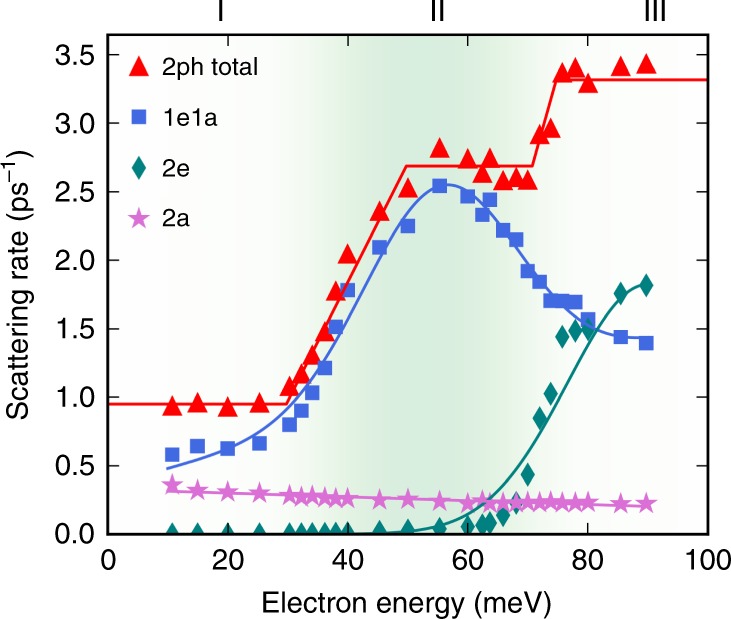
Process-resolved two-phonon scattering rate. Contributions to the 2ph scattering rate in GaAs at 300 K. The scattering rates of the 1e1a, 2e and 2a processes are shown, together with their sum, the total 2ph scattering rate. The solid curves are fits of the data drawn to guide the eye.

**Fig. 6:**
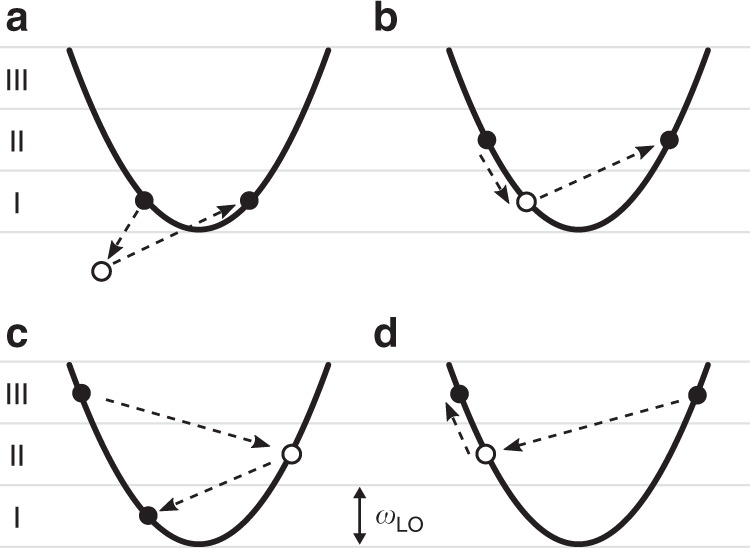
Schematics of two-phonon processes. Schematics of selected 2ph processes in energy region I (**a**), region II (**b**), and region III (**c**, **d**).

The 2ph scattering rate exhibits a trend as a function of energy with three plateaus near the bottom of the conduction band. This trend is a consequence of the dominant *e*–ph interactions in GaAs, which consist of absorption or emission events of LO phonons with energy *ω*_LO_ ≈ 35 meV. To rationalize the energy dependence of the 2ph scattering rate, we define three energy regions, denoted as I, II and III in Figs. 5 and 6, which correspond respectively to electron energies below *ω*_LO_, between *ω*_LO_ and 2*ω*_LO_, and greater than 2*ω*_LO_ referenced to the conduction band minimum, which is taken hereafter to be the electron energy zero. The band structure in all three energy regions is nearly parabolic. We plot the rates of each of the 1e1a, 2e and 2a process contributions in Fig. 5, and sketch the LO phonon emission and absorption processes in each energy region in Fig. 6.

In region I, the electrons possess an energy smaller than *ω*_LO_, and thus cannot emit two LO phonons since this would require a final state in the band gap; the rate of the 2e process is accordingly negligible in region I. The 2a process in which the electron absorbs two LO phonons is active in region I, but it is thermally activated and weak at 300 K since *ω*_LO_ ≈ 35 meV. The 2a process is nearly energy independent, and weak throughout the three regions. The 1e1a process in which an electron absorbs and them emits an LO phonon is the dominant contribution in region I. Recall that the 2ph rate is inversely proportional to the square of the off-shell extent, Δ*E*^2^. The 1e1a channel in which an LO phonon is first emitted and then absorbed is thus suppressed in region I since its intermediate state, resulting from one LO phonon emission, is always off shell, as shown in Fig. 6a.

In region II, the 1e1a scattering rate increases rapidly at energy *ω*_LO_ = 35 meV because the intermediate state accessed after emitting an LO phonon can be on-shell, as shown in Fig. 6b. The rate of the 2e processes remains negligible in region II up to an energy of 2*ω*_LO_. In region III, the contribution from the 2e process increases substantially as the electrons can emit two LO phonons and transition to the bottom of the conduction band through an on-shell process, as shown in Fig. 6c. The 1e1a scattering rate drops from region II to region III due to subtle reasons related to the lifetime of the intermediate state. Recall that for on-shell processes, Δ*E* ≃ 0, and thus the amplitude is proportional to the intermediate state lifetime (see Eq. (5)). An electron in region II can emit a phonon, transition to an on-shell intermediate state in region I, and then absorb another phonon to transition to a final state, as shown in Fig. 6b. As the scattering rates for states in region I are considerably smaller than in the other two regions, the on-shell intermediate states have correspondingly longer lifetimes, thus giving a large amplitude to on-shell 1e1a processes in region II. On the other hand, the 1e1a processes for electrons in region III lead to intermediate states in region II or above (see Fig. 6d), which have much shorter lifetimes than in region I, resulting in a smaller 1e1a rate in region III compared to region II. These arguments allow us to rationalize the 2ph process rates in Fig. 5.

Let us discuss the temperature dependence of the 2ph scattering processes, focusing on the ratio Γ^(2ph)^∕ Γ^(1ph)^ of the 2ph scattering rates to the leading-order 1ph rates. We provide a brief analysis here and a more extensive discussion in the Supplementary Information. The temperature dependence of the 2ph rates originates from the intermediate state lifetimes in the denominators of Eq. (3) and the thermal factors *A*^(*i*)^ in Eq. (4). The intermediate state *e*–ph scattering rate increases with temperature due to an increase in phonon number, so the intermediate state lifetimes become shorter, lowering the 2ph rate Γ^(2ph)^ for increasing temperatures. On the contrary, the thermal factors *A*^(*i*)^, which contain factors proportional to the phonon number *N*, increase rapidly with temperature, making Γ^(2ph)^ greater at higher temperatures. In the 200–400 K temperature range investigated here, these two effects compensate in GaAs, resulting in nearly temperature independent Γ^(2ph)^ ∕ Γ^(1ph)^ ratios. We conclude that the 2ph processes are equally as important relative to the leading-order 1ph processes over a wide temperature range near room temperature.

### Contribution to the electron mobility

**Fig. 7:**
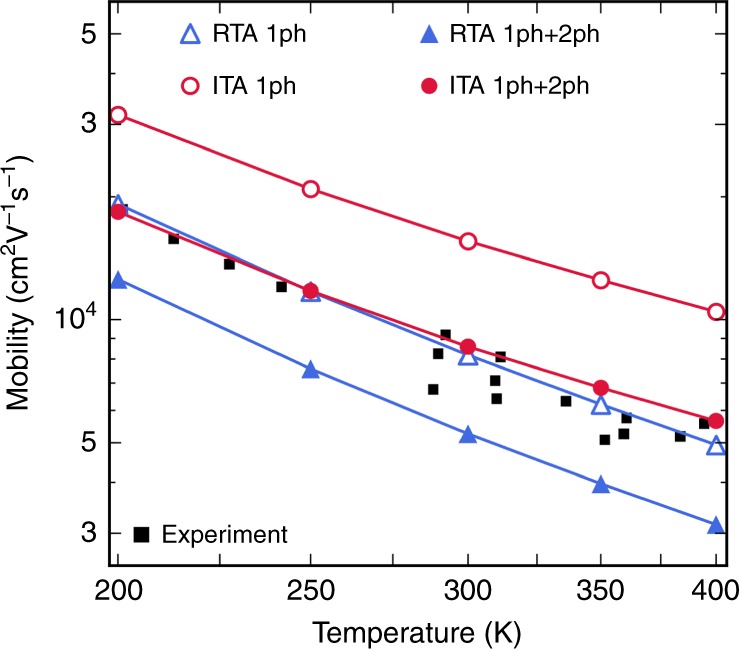
Calculated electron mobility in GaAs. Electron mobility in GaAs, computed by solving the linearized BTE, both within the RTA and with an iterative approach (ITA). For each solution method, two sets of calculations are shown, one that includes only the 1ph leading-order processes and one that includes both 1ph and 2ph scattering. Experimental values are taken from refs. ^33−39^.

Since the 2ph contributions are significant, one expects that they play a role in charge transport. Figure 7 shows the electron mobility in GaAs obtained by solving the BTE both within the relaxation time approximation (RTA) and with a more accurate iterative approach (ITA)^4,7^, which we have extended here to include 2ph processes (see Supplementary Information). Results are given for calculations that either neglect or include the 2ph contributions. The iterative solution with only 1ph scattering overestimates the electron mobility in GaAs by 40–80% at 200–400 K, consistent with the results in ref. ^8^ (and in ref. ^4^, provided the polar correction is not included for the acoustic modes, which artificially increases the acoustic mode contribution to scattering in ref. ^4^ and lowers the computed mobility). This result is puzzling, since the BTE can accurately predict the mobility in nonpolar semiconductors; the discrepancy with experiment is too large to be due to small errors in the electron effective mass (in our calculation, the effective mass is 0.055*m*_0_ versus an experimental value of 0.067*m*_0_, where *m*_0_ is the electron mass). Redoing the mobility calculation with a band structure with the experimental effective mass of 0.067*m*_0_ (see Supplementary Fig. 3) reduces the mobility overestimate to roughly 20% compared to experiment for the iterative BTE solution with 1ph scattering, and including 2ph processes still improves the agreement with experiment of the mobility and its temperature dependence.

This open problem is solved here by including the 2ph processes in the ITA (see Fig. 7), which lowers the mobility due to the additional 2ph scattering and gives mobility values in excellent agreement with experiments^32–38^. Both the absolute value of the mobility and its temperature dependence improve when the 2ph processes are included. This result implies that the agreement with experiment of the RTA with 1ph processes^3^ is due to error compensation. We conclude that the 2ph contributions are crucial to improving the computed electron mobility in GaAs.

Since the long-range LO mode coupling is dominant in many polar semiconductors and oxides, we expect the 2ph processes to be important in a wide range of polar materials. We apply our approach to BaSnO_3_, a weakly polar oxide with dominant LO-mode *e*–ph interactions and a high room-temperature electron mobility. We compute the 2ph scattering rates and the mobility including 2ph processes in BaSnO_3_ (see Supplementary Fig. 2). Similar to GaAs, we find that the mobility computed with the iterative BTE including only 1ph processes is substantially higher than the experimental value, while adding 2ph scattering significantly improves the agreement with experiment. This result confirms that our approach is broadly applicable to weakly polar semiconductors and oxides, in which perturbation theory is valid and the mobility near room temperature is usually limited by scattering with the LO mode.

## Discussion

Our calculations show quantitatively that the 2ph contributions are substantial even in a weakly polar material such as GaAs in which higher-order *e*–ph interactions and polaron effects are typically neglected. The approach introduced in this work is general, and it is expected to give accurate *e*–ph scattering rates and carrier mobilities in weakly polar III-V and II-VI semiconductors and in polar oxides with high room-temperature mobility (and thus, weak polaron effects^20^); the perturbative approach is valid in these materials, where the long-range *e*–ph interactions make next-to-leading-order effects substantial.

**Fig. 8:**
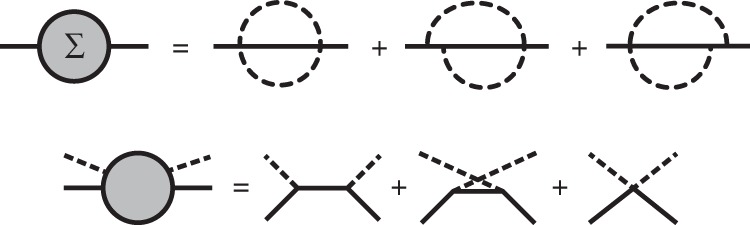
Diagrams beyond this work. The top panel shows some of the additional self-energy diagrams resulting from the four-point Debye−Waller vertex, which introduces a new 1e1a process interfering with the two considered in this work in the bottom panel.

An open question left out for future work is whether the 2ph processes due to acoustic modes can be important in metals and nonpolar materials. A practical observation is that the mobility computed using only 1ph processes matches experiment closely in elemental metals^2^ and nonpolar semiconductors^8^; therefore, 2ph acoustic processes are expected to be negligible, although a rigorous proof (or numerical evidence) of this point is more challenging. As discussed by Kocevar^12^, there are two *e*–ph perturbations contributing to 2ph processes. The one considered in this work is due to the first derivative of the potential with respect to lattice vibrations, taken to second order in perturbation theory, which leads to 2ph scattering consisting of two consecutive 1ph scattering events. The second contribution, which is not included in this work, is due to the second derivatives of the potential with respect to lattice vibrations; it leads to a direct 2ph interaction associated with the so-called Debye-Waller (DW) vertex, which represents an electron interacting simultaneously with two phonons^12^. The DW vertex leads to additional self-energy diagrams and 2ph processes, some of which are illustrated in Fig. 8.

Due to translational invariance, for acoustic phonons in the long-wavelength limit there is a strong cancellation between the scattering processes considered here and those due to the DW interaction^12,39^. This cancellation has also been hypothesized to be valid for acoustic phonons beyond the long-wavelength limit, although this point has never been proven. This result, together with the numerical evidence that 1ph processes are sufficient to explain the mobility in nonpolar semiconductors and elemental metals, is suggestive of a negligible role of 2ph acoustic processes. Since the DW vertex cannot currently be computed ab initio for two general phonon momenta, numerical studies of 2ph processes due to the DW interaction are left out for future work. In the calculations on the two weakly polar materials considered here, GaAs and BaSnO_3_, the LO mode interaction is dominant, and our results are nearly unchanged if acoustic phonons are included (see Supplementary Fig. 1).

In summary, our calculations of the 2ph scattering rates and their contribution to the mobility pave the way to studying higher-order *e*–ph interactions and charge transport in polar materials from first principles. Together with recently proposed methods to treat charge transport in materials with polarons and stronger *e*–ph interactions^24^, it is clear that ab initio calculations are becoming able to investigate *e*–ph interactions and charge transport in a wide range of polar materials.

## Methods

### Numerical calculations

The DFT and lowest-order *e*–ph calculations in GaAs follow our previous work^3^. Briefly, we carry out DFT calculations in GaAs using the Quantum ESPRESSO code^40^ with a plane-wave basis set. We employ the local density approximation^41^ and norm-conserving pseudopotentials^42^. A relaxed lattice constant of 5.55 Å, a kinetic energy cutoff of 72 Ry and 8 × 8 × 8 **k**-point grids are used in all DFT calculations. Phonon dispersions are computed with density functional perturbation theory (DFPT)^43^ on an 8 × 8 × 8 **q**-point grid. The *e*–ph coupling constants, *g*_*nmν*_(**k**, **q**), are computed using these coarse **k**- and **q**-point grids^3^ using DFPT together with our in-house developed Perturbo code^31^, and interpolated using Wannier functions^44^ generated with Wannier90^45^.

To compute and converge the 2ph scattering rates, we use Monte Carlo integration by sampling up to 3 billion random (**q**, **p**) pairs of Brillouin zone points drawn from a Cauchy distribution^3^. The delta function in Eq. (2) is approximated by a Gaussian with a small broadening of 5 meV. Since in GaAs the LO phonon dominates *e*–ph scattering for electrons within  ~100 meV of the conduction band edge^3^, in the 2ph scattering rate calculations we use only the long-range coupling to the LO modes^11,46,47^, which greatly reduces the 2ph computational cost while giving 2ph scattering rates nearly identical to calculations that include all phonon modes (see Supplementary Fig. 1). We use a carrier concentration of 10^16^ cm^−3^ in all scattering rate and mobility calculations, and set the chemical potential accordingly at each temperature. The mobility calculations are carried out by integrating over energies of up to 250 meV above the conduction band minimum; we have verified that this energy window is sufficient to converge the mobility.

We highlight that the 2ph scattering rate calculations are very computationally expensive due to the large number of points sampled in the BZ integral over two-phonon momenta. The average cost for computing the 2ph scattering rate in GaAs is  ~500 CPU core hours per electronic state for each iteration over the intermediate state lifetime; a typical calculation requires ten iterations to converge, for a total cost of 5000 CPU core hours per electronic state. By contrast, it takes only 0.08 CPU core hours (5 min on a single CPU core) per electronic state to converge the 1ph scattering rate using our code. The computational cost of the 2ph scattering rates is thus approximately 10^4^−10^5^ higher than the 1ph scattering rates.

## Supplementary information

Supplementary Information

## Data Availability

The data that support the findings of this study are available from the corresponding author upon reasonable request.

## Code availability

The code employed in this work, Perturbo, is available for download at https://perturbo-code.github.io/. The 2ph routines developed in this work are available from the corresponding author upon reasonable request.

## References

[1] Bernardi M (2016). First-principles dynamics of electrons and phonons. Eur. Phys. J. B.

[2] Mustafa JI, Bernardi M, Neaton JB, Louie SG (2016). Ab initio electronic relaxation times and transport in noble metals. Phys. Rev. B.

[3] Zhou J-J, Bernardi M (2016). Ab initio electron mobility and polar phonon scattering in GaAs. Phys. Rev. B.

[4] Liu T-H, Zhou J, Liao B, Singh DJ, Chen G (2017). First-principles mode-by-mode analysis for electron-phonon scattering channels and mean free path spectra in GaAs. Phys. Rev. B.

[5] Zhou J-J, Hellman O, Bernardi M (2018). Electron-phonon scattering in the presence of soft modes and electron mobility in SrTiO_3_ perovskite from first principles. Phys. Rev. Lett..

[6] Lee N-E, Zhou J-J, Agapito LA, Bernardi M (2018). Charge transport in organic molecular semiconductors from first principles: the bandlike hole mobility in a naphthalene crystal. Phys. Rev. B.

[7] Li W (2015). Electrical transport limited by electron-phonon coupling from Boltzmann transport equation: an ab initio study of Si, Al, and MoS_2_. Phys. Rev. B.

[8] Ma J, Nissimagoudar AS, Li W (2018). First-principles study of electron and hole mobilities of Si and GaAs. Phys. Rev. B.

[9] Sohier T, Campi D, Marzari N, Gibertini M (2018). Mobility of two-dimensional materials from first principles in an accurate and automated framework. Phys. Rev. Mater..

[10] Fröhlich H (1954). Electrons in lattice fields. Adv. Phys..

[11] Vogl P (1976). Microscopic theory of electron-phonon interaction in insulators or semiconductors. Phys. Rev. B.

[12] Kocevar, P. *Multiphonon Scattering* 167–174 (Springer US, 1980).

[13] Alldredge GP, Blatt FJ (1967). On the role of two-phonon processes in the energy relaxation of a heated-electron distribution. Ann. Phys..

[14] Sher A, Thornber KK (1967). Resonant electron-phonon scattering in polar semiconductors. Appl. Phys. Lett..

[15] Thorbergsson GI, Sak J (1986). Mobility of an acoustic polaron at very low temperatures. Phys. Lett. A.

[16] Mora-Ramos ME, Rodríguez FJ, Quiroga L (1999). Polaron properties of III-V nitride compounds: second-order effects. J. Phys. Condens. Matter.

[17] Smondyrev MA (1986). Diagrams in the polaron model. Theor. Math. Phys..

[18] Wang Z, Mahan GD (1989). Low-temperature resistivity from electron-dual-phonon processes for alkali metals. Phys. Rev. B.

[19] Woods LM, Mahan GD (1998). Nonlinear electron-phonon heat exchange. Phys. Rev. B.

[20] Emin, D. *Polarons* (Cambridge University Press, 2012).

[21] Gunnarsson O, Meden V, Schönhammer K (1994). Corrections to Migdal’s theorem for spectral functions: a cumulant treatment of the time-dependent Green’s function. Phys. Rev. B.

[22] Story SM, Kas JJ, Vila FD, Verstraete MJ, Rehr JJ (2014). Cumulant expansion for phonon contributions to the electron spectral function. Phys. Rev. B.

[23] Nery JP (2018). Quasiparticles and phonon satellites in spectral functions of semiconductors and insulators: cumulants applied to the full first-principles theory and the Fröhlich polaron. Phys. Rev. B.

[24] Zhou J-J, Bernardi M (2019). Predicting charge transport in the presence of polarons: the beyond-quasiparticle regime in SrTiO_3_. Phys. Rev. Res..

[25] Noffsinger J, Kioupakis E, Van de Walle CG, Louie SG, Cohen ML (2012). Phonon-assisted optical absorption in silicon from first principles. Phys. Rev. Lett..

[26] Murayama M, Nakayama T (1995). Ab initio calculations of two-photon absorption spectra in semiconductors. Phys. Rev. B.

[27] Hayat A, Ginzburg P, Orenstein M (2008). Observation of two-photon emission from semiconductors. Nat. Photonics.

[28] Bern Z, Dixon L, Ghinculov A (2001). Two-loop correction to Bhabha scattering. Phys. Rev. D.

[29] Aoyama T, Kinoshita T, Nio M (2018). Revised and improved value of the QED tenth-order electron anomalous magnetic moment. Phys. Rev. D.

[30] Mahan, G. D. *Many-Particle Physics*, 3rd edn (Springer, 2000).

[31] Zhou, J.-J. et al. Perturbo: a software package for *ab initio* electron-phonon interactions, charge transport and ultrafast dynamics. Preprint at https://arxiv.org/abs/2002.02045 (2020).

[32] Rode DL (1970). Electron mobility in direct-gap polar semiconductors. Phys. Rev. B.

[33] Rode DL, Knight S (1971). Electron transport in GaAs. Phys. Rev. B.

[34] Blakemore JS (1982). Semiconducting and other major properties of gallium arsenide. J. Appl. Phys..

[35] Hicks HGB, Manley DF (1969). High purity GaAs by liquid phase epitaxy. Solid State Commun..

[36] Wolfe CM, Stillman GE, Lindley WT (1970). Electron mobility in high-purity GaAs. J. Appl. Phys..

[37] Blood P (1972). Electrical properties of *n*-type epitaxial GaAs at high temperatures. Phys. Rev. B.

[38] Nichols KH, Yee CML, Wolfe CM (1980). High-temperature carrier transport in n-type epitaxial GaAs. Solid-State Electron..

[39] Holstein T (1959). Theory of ultrasonic absorption in metals: the collision-drag effect. Phys. Rev..

[40] Giannozzi P (2009). Quantum ESPRESSO: a modular and open-source software project for quantum simulations of materials. J. Phys. Condens. Matter.

[41] Perdew JP, Zunger A (1981). Self-interaction correction to density-functional approximations for many-electron systems. Phys. Rev. B.

[42] Troullier N, Martins JL (1991). Efficient pseudopotentials for plane-wave calculations. Phys. Rev. B.

[43] Baroni S, de Gironcoli S, DalCorso A, Giannozzi P (2001). Phonons and related crystal properties from density-functional perturbation theory. Rev. Mod. Phys..

[44] Giustino F, Cohen ML, Louie SG (2007). Electron-phonon interaction using Wannier functions. Phys. Rev. B.

[45] Mostofi AA (2014). An updated version of wannier90: A tool for obtaining maximally-localised Wannier functions. Comput. Phys. Commun..

[46] Sjakste J, Vast N, Calandra M, Mauri F (2015). Wannier interpolation of the electron-phonon matrix elements in polar semiconductors: polar-optical coupling in GaAs. Phys. Rev. B.

[47] Verdi C, Giustino F (2015). Fröhlich electron-phonon vertex from first principles. Phys. Rev. Lett..

