# Supplementary material for: Ab initio electron-two-phonon scattering in GaAs from next-to-leading order perturbation theory
Source: Nat Commun. 2020 Mar 30;11:1607. doi: 10.1038/s41467-020-15339-0 (PMC7105459; doi:10.1038/s41467-020-15339-0)
Supplement: Supplementary file 1 — Supplementary Information [file 41467_2020_15339_MOESM1_ESM.pdf]

Supplementary Information for "*Ab Initio* Electron-Two-Phonon Scattering in GaAs from Next-to-Leading Order Perturbation Theory"

Nien-En Lee,[1,2] Jin-Jian Zhou,[1] Hsiao-Yi Chen,[1,2] and Marco Bernardi[1]

[1] *Department of Applied Physics and Materials Science, California Institute of Technology, Pasadena, California 91125, USA*

[2] *Department of Physics, California Institute of Technology, Pasadena, California 91125, USA*

Supplementary Figure 1

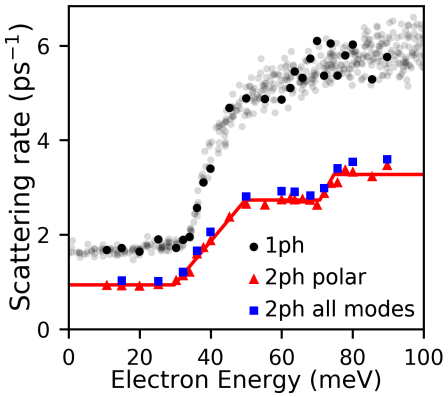

Supplementary Figure 1. **Two-phonon scattering rates with all phonon modes included.** The 2ph scattering rates $\Gamma^{(2ph)}_{n\mathbf{k}}$ in GaAs at 300 K, computed using only the LO mode (curve labelled "2ph polar") and with all phonon modes included (curve labelled "2ph all modes").

Supplementary Figure 2

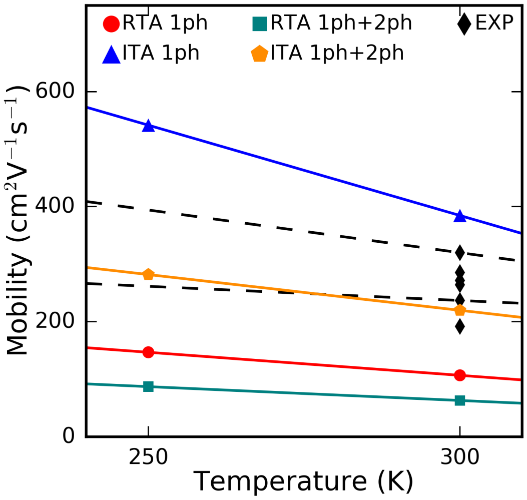

Supplementary Figure 2. **Mobility calculation in $BaSnO_3$.** Electron mobility in $BaSnO_3$ computed using the same methods as in Fig. 7 of the main text. It is seen that the iterative BTE with only 1ph scattering overestimates the mobility, while including 2ph scattering improves the agreement with experiment, similar to GaAs. Experimental data are shown with markers at room temperature and dashed lines at lower temperatures. The experimental data are from Figs. 4 and 5 in Ref. [1]. We used the single crystal mobility data for a $0.5 - 1.5 \times 10^{20}$ cm^{-3} carrier concentration range. Our calculation used a 0.8×10^{20} cm^{-3} carrier concentration.

Supplementary Figure 3

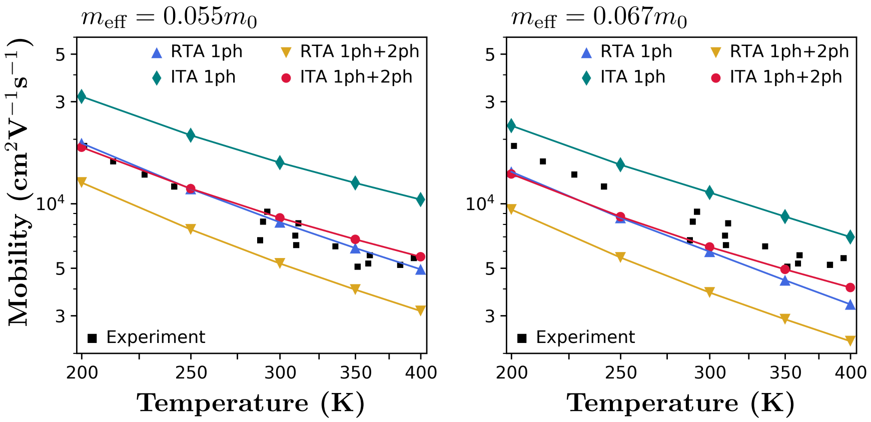

Supplementary Figure 3. **Mobility results with experimental effective mass.** Comparison of mobility results calculated from our DFT band structure with effective mass of $0.055m_0$ (left panel) and a band structure with the experimental effective mass of $0.067m_0$ (right panel) obtained by manually rescaling the DFT eigenvalues by a factor of 0.067/0.055. Here, m_0 is the bare electron mass.

Supplementary Note 1: Analytic Derivation of the Scattering Rates of Two-Phonon Processes

In this section, we calculate the contributions to the electron-phonon (e-ph) scattering rates from the next-to-leading-order self-energy diagrams. We use the Matsubara technique to calculate the two-loop self-energy, whose imaginary part is related to the total scattering rates via the optical theorem (see Supplementary Figure 4). We focus on the scattering processes with two external phonons.

The Feynmann rules, which have been derived in Mahan [2], will be adapted here to our context. The starting point is the e-ph Hamiltonian

$$H = \sum_{n\mathbf{k}} \varepsilon_{n\mathbf{k}} a^\dagger_{n\mathbf{k}} a_{n\mathbf{k}} + \sum_{\nu\mathbf{q}} \hbar\omega_{\nu\mathbf{q}} \left(b^\dagger_{\nu\mathbf{q}} b_{\nu\mathbf{q}} + \frac{1}{2} \right) + \frac{1}{\sqrt{N_\Omega}} \sum_{mn\mathbf{k}} \sum_{\nu\mathbf{q}} g_{mn\nu}(\mathbf{k},\mathbf{q}) \left(b^\dagger_{\nu-\mathbf{q}} + b_{\nu\mathbf{q}} \right) a^\dagger_{m\mathbf{k}+\mathbf{q}} a_{n\mathbf{k}},$$

where $a_{n\mathbf{k}}$ and $b_{\nu\mathbf{q}}$ are the annihilation operators for electrons and phonons with energies $\varepsilon_{n\mathbf{k}}$ and $\hbar\omega_{\nu\mathbf{q}}$, respectively, $g_{mn\nu}(\mathbf{k},\mathbf{q})$ is the e-ph coupling constant, and N_Ω is the number of unit cells in the crystal. Comparing with Eqs. (2.67) and (3.200) in Ref. [2], we have introduced the dependence on electron crystal momentum $\mathbf{k}$ for the e-ph couplings and the electron band indices m and n. Also note that for the Hamiltonian to be Hermitian, the e-ph couplings must satisfy $g^*_{mn\nu}(\mathbf{k},\mathbf{q}) = g_{nm\nu}(\mathbf{k}+\mathbf{q},-\mathbf{q})$.

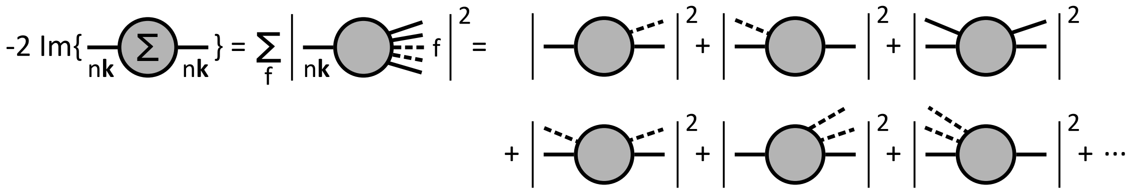

Supplementary Figure 4. **Schematic of the scattering rate series.** The first equality is the optical theorem that relates the imaginary part of the self-energy to the total scattering rates. Here and below, solid and dashed lines represent electron and phonon propagators, respectively. The three processes in the last row are the two-phonon processes on which we will be focusing.

Feynmann Rules

The rules for constructing diagrams are listed in Sec. 3.4 of Ref. [2]. We slightly modify them here:

1. With each internal electron line, associate the propagator $\mathcal{G}^{(0)}(\mathbf{k}, ik_\lambda) = 1/(ik_\lambda - \xi_{n\mathbf{k}})$, where $\xi_{n\mathbf{k}} = \varepsilon_{n\mathbf{k}} - \mu$ and μ is the chemical potential.

2. With each internal phonon line, associate the propagator $\mathcal{D}^{(0)}(\mathbf{q}, i\omega_\kappa) = -2\omega_{\nu\mathbf{q}}/(\omega_\kappa^2 + \omega_{\nu\mathbf{q}}^2)$. Note that we set $\hbar = 1$ for convenience here and below; it can easily be restored by dimensional analysis.
3. With each vertex, associate the e-ph coupling constant $g_{mn\nu}(\mathbf{k}, \mathbf{q})$. Beware of the direction of $\mathbf{q}$.
4. Conserve momentum and complex frequency at each vertex and sum over the internal degrees of freedom.
5. Multiply the expression by

$$\frac{(-1)^{L+F}(2S+1)^F}{(\beta N_\Omega)^L} = \left(-\frac{1}{\beta N_\Omega}\right)^L (-2)^F\,,$$

where F is the number of closed Fermion loops. The $(2S+1)$ factor is a summation over spin degrees of freedom, and $2S+1 = 2$ for electrons. The integer L is the number of loops, and $\beta = 1/k_B T$, where T is temperature.

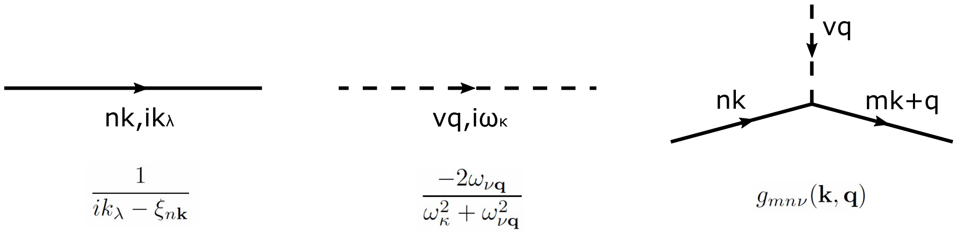

Electron Self-Energy

We consider below the 1-loop diagram that gives the lowest-order (one-phonon) self-energy and the three relevant two-loop diagrams for the electron self-energy. Diagram IIc will not contribute to the two-phonon processes and thus will not be considered in the following.

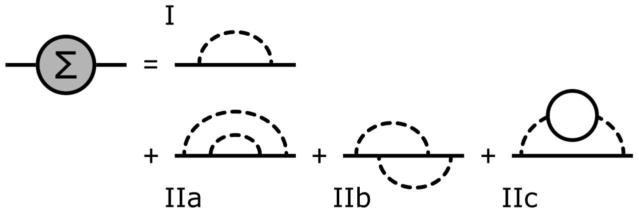

One-Loop Diagram I

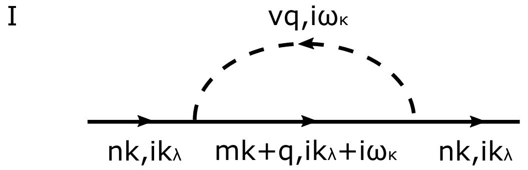

As a warm up exercise, we first derive the one-loop self-energy diagram, labelled as I in figure above. Since $L = 1$ and $F = 0$, the Feynman rules give

$$\Sigma^{(\mathrm{I})} = -\frac{1}{\beta N_\Omega}\sum_{m\nu\mathbf{q}}\sum_{i\omega_\kappa} g_{mn\nu}(\mathbf{k}, \mathbf{q})\mathcal{D}^{(0)}(\mathbf{q}, i\omega_\kappa)\mathcal{G}^{(0)}(\mathbf{k}+\mathbf{q}, ik_\lambda + i\omega_\kappa)g_{nm\nu}(\mathbf{k}+\mathbf{q}, -\mathbf{q})$$

$$= -\frac{1}{\beta N_\Omega}\sum_{m\nu\mathbf{q}}\sum_{i\omega_\kappa}|g_{mn\nu}(\mathbf{k},\mathbf{q})|^2\frac{-2\omega_{\nu\mathbf{q}}}{\omega_\kappa^2+\omega_{\nu\mathbf{q}}^2}\frac{1}{ik_\lambda+i\omega_\kappa-\xi_{m\mathbf{k}+\mathbf{q}}}$$
$$\equiv \frac{1}{N_\Omega}\sum_{m\nu\mathbf{q}}|g_{mn\nu}(\mathbf{k},\mathbf{q})|^2\left(-\frac{1}{\beta}\right)\sum_{i\omega_\kappa}f(i\omega_\kappa),$$

where $f(z)$ is defined as

$$f(z)\equiv\frac{2\omega_{\nu\mathbf{q}}}{z^2-\omega_{\nu\mathbf{q}}^2}\frac{1}{z+ik_\lambda-\xi_{m\mathbf{k}+\mathbf{q}}}.$$

To apply the Matsubara frequency summation method, we define the bosonic weighting function as in [2]:

$$n_B(z)=\frac{1}{e^{\beta z}-1},$$

whose poles are at $i\omega_\kappa = i2\kappa\pi/\beta$, with residues $1/\beta$ and integer κ values. The weighting function for fermions is

$$n_F(z)=\frac{1}{e^{\beta z}+1},$$

whose poles are at $ik_\lambda = i(2\lambda+1)\pi/\beta$, with residues $-1/\beta$ and integer λ values.

For this diagram, we will do the contour integral for $f(z)n_B(z)$ at the complex infinity. Since $f(z)n_B(z)$ decays faster than $1/z$, we can apply the Cachy residue theorem, which gives (here and below, z' are the relevant poles):

$$\begin{aligned}0 &= \lim_{|z|\to\infty}\oint\frac{dz}{2\pi i}f(z)n_B(z)\\ &= \sum_{z'\text{ of }f\cdot n_B}\mathrm{Res}\{f(z')n_B(z')\}\\ &= \sum_{i\omega_\kappa}f(i\omega_\kappa)\frac{1}{\beta}+\sum_{z'\text{ of }f}\mathrm{Res}\{f(z')n_B(z')\}.\end{aligned}$$

Using this result, we get:

$$\Sigma^{(\mathrm{I})}=\frac{1}{N_\Omega}\sum_{m\nu\mathbf{q}}|g_{mn\nu}(\mathbf{k},\mathbf{q})|^2\sum_{z'\text{ of }f}\mathrm{Res}\{f(z')\}n_B(z').$$

The poles of $f(z)$ are at $z_1=\omega_{\nu\mathbf{q}}$, $z_2=-\omega_{\nu\mathbf{q}}$ and $z_3=-ik_\lambda+\xi_{m\mathbf{k}+\mathbf{q}}$. Their residues are

$$\begin{aligned}\mathrm{Res}\{f(z),z_1\} &= \frac{1}{\omega_{\nu\mathbf{q}}+ik_\lambda-\xi_{m\mathbf{k}+\mathbf{q}}}\\ \mathrm{Res}\{f(z),z_2\} &= -\frac{1}{-\omega_{\nu\mathbf{q}}+ik_\lambda-\xi_{m\mathbf{k}+\mathbf{q}}}\\ \mathrm{Res}\{f(z),z_3\} &= \frac{2\omega_{\nu\mathbf{q}}}{(-ik_\lambda+\xi_{m\mathbf{k}+\mathbf{q}})^2-\omega_{\nu\mathbf{q}}^2}=\frac{1}{-\omega_{\nu\mathbf{q}}+ik_\lambda-\xi_{m\mathbf{k}+\mathbf{q}}}-\frac{1}{\omega_{\nu\mathbf{q}}+ik_\lambda-\xi_{m\mathbf{k}+\mathbf{q}}}.\end{aligned}$$

We also know that $n_B(z_1)=n_B(\omega_{\nu\mathbf{q}})\equiv N_{\nu\mathbf{q}}$, $n_B(z_2)=n_B(-\omega_{\nu\mathbf{q}})=-N_{\nu\mathbf{q}}-1$ and $n_B(z_3)=-n_F(\xi_{m\mathbf{k}+\mathbf{q}})\equiv -f_{m\mathbf{k}+\mathbf{q}}$, where N and f are the thermal occupation numbers for phonons and electrons, respectively. We also used the fact that $ik_\lambda = i(2\lambda+1)\pi/\beta$. Substituting this result in the self-energy expression, we get

$$\Sigma^{(\mathrm{I})}=\frac{1}{N_\Omega}\sum_{m\nu\mathbf{q}}|g_{mn\nu}(\mathbf{k},\mathbf{q})|^2\left[\frac{N_{\nu\mathbf{q}}+f_{m\mathbf{k}+\mathbf{q}}}{ik_\lambda+\omega_{\nu\mathbf{q}}-\xi_{m\mathbf{k}+\mathbf{q}}}+\frac{1+N_{\nu\mathbf{q}}-f_{m\mathbf{k}+\mathbf{q}}}{ik_\lambda-\omega_{\nu\mathbf{q}}-\xi_{m\mathbf{k}+\mathbf{q}}}\right].$$

Employing the analytic continuation $ik_\lambda\to E+i\eta$, we obtain the off-shell lowest-order e-ph self-energy:

$$\Sigma^{(\mathrm{I})}(E)=\frac{1}{N_\Omega}\sum_{m\nu\mathbf{q}}|g_{mn\nu}(\mathbf{k},\mathbf{q})|^2\left[\frac{N_{\nu\mathbf{q}}+f_{m\mathbf{k}+\mathbf{q}}}{E+\omega_{\nu\mathbf{q}}-\xi_{m\mathbf{k}+\mathbf{q}}+i\eta}+\frac{1+N_{\nu\mathbf{q}}-f_{m\mathbf{k}+\mathbf{q}}}{E-\omega_{\nu\mathbf{q}}-\xi_{m\mathbf{k}+\mathbf{q}}+i\eta}\right].$$

We will be mainly interested in the scattering rate at the electron energy $\xi_{n\mathbf{k}}$ and therefore we will set $E = \xi_{n\mathbf{k}}$ to obtain the on-shell self-energy for the state with band n and crystal momentum $\mathbf{k}$. Using the identity

$$\frac{1}{x + i\eta} = P\frac{1}{x} - i\pi\delta(x)$$

and Eq. (7.304) in Ref. [2], which states that the scattering rate Γ is obtained as $\Gamma = -(2/\hbar)\mathrm{Im}\Sigma$, we get

$$\Gamma^{(\mathrm{I})}_{n\mathbf{k}} = \frac{2\pi}{\hbar}\frac{1}{N_\Omega}\sum_{m\nu\mathbf{q}} |g_{mn\nu}(\mathbf{k},\mathbf{q})|^2 \left[(N_{\nu\mathbf{q}} + f_{m\mathbf{k}+\mathbf{q}})\delta(\xi_{n\mathbf{k}} + \hbar\omega_{\nu\mathbf{q}} - \xi_{m\mathbf{k}+\mathbf{q}}) + \{(1 + N_{\nu\mathbf{q}} - f_{m\mathbf{k}+\mathbf{q}})\delta(\xi_{n\mathbf{k}} - \hbar\omega_{\nu\mathbf{q}} - \xi_{m\mathbf{k}+\mathbf{q}})\right],$$

with $\hbar$ placed back into the expression. This is the well-known lowest-order scattering rate commonly used in first-principles calculations.

Two-Loop Diagram IIa

Now we compute the first two-loop diagram, which is shown in the figure below. Since $L = 2$ and $F = 0$, the Feynman rules give

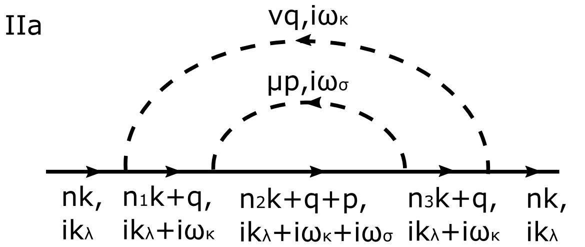

$$\Sigma^{(\mathrm{IIa})} = \frac{1}{\beta^2 N_\Omega^2}\sum_{n_1n_2n_3}\sum_{\nu\mathbf{q}}\sum_{\mu\mathbf{p}} g_{n_1n\nu}(\mathbf{k},\mathbf{q})g^*_{n_3n\nu}(\mathbf{k},\mathbf{q})g_{n_2n_1\mu}(\mathbf{k}+\mathbf{q},\mathbf{p})g^*_{n_2n_3\mu}(\mathbf{k}+\mathbf{q},\mathbf{p})\sum_{i\omega_\kappa}\sum_{i\omega_\sigma} f(i\omega_\kappa, i\omega_\sigma),$$

where $f(i\omega_\kappa, i\omega_\sigma)$ is defined as

$$\begin{aligned} f(i\omega_\kappa, i\omega_\sigma) &\equiv \frac{1}{ik_\lambda + i\omega_\kappa - \xi_{n_1\mathbf{k}+\mathbf{q}}}\,\frac{1}{ik_\lambda + i\omega_\kappa + i\omega_\sigma - \xi_{n_2\mathbf{k}+\mathbf{q}+\mathbf{p}}}\,\frac{1}{ik_\lambda + i\omega_\kappa - \xi_{n_3\mathbf{k}+\mathbf{q}}}\,\frac{2\omega_{\nu\mathbf{q}}}{\omega_\kappa^2 + \omega_{\nu\mathbf{q}}^2}\,\frac{2\omega_{\mu\mathbf{p}}}{\omega_\sigma^2 + \omega_{\mu\mathbf{p}}^2} \\ &\equiv A_\kappa\,\frac{1}{ik_\lambda + i\omega_\kappa + i\omega_\sigma - \xi_{n_2\mathbf{k}+\mathbf{q}+\mathbf{p}}}\,\frac{2\omega_{\mu\mathbf{p}}}{-(i\omega_\sigma)^2 + \omega_{\mu\mathbf{p}}^2}, \end{aligned}$$

where in A_κ we collect all terms independent of $i\omega_\sigma$. Let us sum over $i\omega_\sigma$ first. Performing the contour integral for $f(i\omega_\kappa, z)n_B(z)$ gives

$$\sum_{i\omega_\sigma} f(i\omega_\kappa, i\omega_\sigma) = -\beta\sum_{z' \text{ of } f}\mathrm{Res}\{f(i\omega_\kappa, z')n_B(z')\}.$$

Note that $f(i\omega_\kappa, z)$ has three poles, whose residues and bosonic weight factors are computed as:

$$\begin{aligned} z_1 &\rightarrow -ik_\lambda - i\omega_\kappa + \xi_{n_2\mathbf{k}+\mathbf{q}+\mathbf{p}} \\ \mathrm{Res}\{f(i\omega_\kappa, z_1)\} &= A_\kappa\left(\frac{1}{z_1 + \omega_{\mu\mathbf{p}}} - \frac{1}{z_1 - \omega_{\mu\mathbf{p}}}\right) \\ n_B(z_1) &= -f_{n_2\mathbf{k}+\mathbf{q}+\mathbf{p}} \end{aligned}$$

$$z_2 \rightarrow \omega_{\mu\mathbf{p}}$$

$$
\begin{aligned}
\mathrm{Res}\{f(i\omega_\kappa, z_2)\} &= -A_\kappa \frac{1}{z_2 + ik_\lambda + i\omega_\kappa - \xi_{n_2\mathbf{k}+\mathbf{q}+\mathbf{p}}} \\
n_B(z_2) &= N_{\mu\mathbf{p}}
\end{aligned}
$$

$$
\begin{aligned}
z_3 &\to -\omega_{\mu\mathbf{p}} \\
\mathrm{Res}\{f(i\omega_\kappa, z_3)\} &= A_\kappa \frac{1}{z_3 + ik_\lambda + i\omega_\kappa - \xi_{n_2\mathbf{k}+\mathbf{q}+\mathbf{p}}} \\
n_B(z_3) &= -N_{\mu\mathbf{p}} - 1
\end{aligned}
$$

Using these results, we get

$$
\sum_{i\omega_\sigma} f(i\omega_\kappa, i\omega_\sigma) = \beta A_\kappa \frac{1 + N_{\mu\mathbf{p}} - f_{n_2\mathbf{k}+\mathbf{q}+\mathbf{p}}}{ik_\lambda + i\omega_\kappa - \xi_{n_2\mathbf{k}+\mathbf{q}+\mathbf{p}} - \omega_{\mu\mathbf{p}}} + \beta A_\kappa \frac{N_{\mu\mathbf{p}} + f_{n_2\mathbf{k}+\mathbf{q}+\mathbf{p}}}{ik_\lambda + i\omega_\kappa - \xi_{n_2\mathbf{k}+\mathbf{q}+\mathbf{p}} + \omega_{\mu\mathbf{p}}} \equiv \beta h^{(-)}(i\omega_\kappa) + \beta h^{(+)}(i\omega_\kappa),
$$

where we defined two functions, $h^{(-)}(i\omega_\kappa)$ and $h^{(+)}(i\omega_\kappa)$, as the first and second terms in the expression above.

We then sum over $i\omega_\kappa$, using again

$$
\sum_{i\omega_\kappa} h^{(\pm)}(i\omega_\kappa) = -\beta \sum_{z' \text{ of } h^{(\pm)}} \mathrm{Res}\{h^{(\pm)}(z') n_B(z')\}.
$$

A subtle point is that the cases with $n_3 \neq n_1$ and $n_3 = n_1$ have different pole structures, and need to be discussed separately (see the figure above for diagram IIa; n_3 and n_1 label two intermediate electronic states in the self-energy diagram). Luckily, the two cases give the same expression for the two-phonon scattering processes, as we show explicitly below. Before carrying out the calculation, let us introduce some useful abbreviations. We will use in the following $\xi_{n_1\mathbf{k}+\mathbf{q}} \equiv \xi_1$, $\xi_{n_1\mathbf{k}+\mathbf{p}} \equiv \xi_{1\mathbf{p}}$, $f_{n_2\mathbf{k}+\mathbf{q}+\mathbf{p}} \equiv f_2$, $\omega_{\nu\mathbf{q}} \equiv \omega_\mathbf{q}$, etc.

Case with $n_3 \neq n_1$

Let us focus on $h^{(-)}$ for the case $n_3 \neq n_1$ first. In this case, $h^{(-)}$ is defined as

$$
h^{(-)}(z) = \frac{1}{z + ik_\lambda - \xi_1} \, \frac{1}{z + ik_\lambda - \xi_3} \, \frac{-2\omega_\mathbf{q}}{z^2 - \omega_\mathbf{q}^2} \, \frac{1 + N_\mathbf{p} - f_2}{z + ik_\lambda - \xi_2 - \omega_\mathbf{p}}.
$$

It has five poles, which are given here together with their residues and bosonic weight factors:

$$
\begin{aligned}
z_1 &\to -ik_\lambda + \xi_1 \\
\mathrm{Res}\{h^{(-)}(z_1)\} &= \frac{1}{\xi_1 - \xi_3} \, \frac{1 + N_\mathbf{p} - f_2}{\xi_1 - \xi_2 - \omega_\mathbf{p}} \left(\frac{1}{-ik_\lambda + \xi_1 + \omega_\mathbf{q}} - \frac{1}{-ik_\lambda + \xi_1 - \omega_\mathbf{q}} \right) \\
n_B(z_1) &= -f_1
\end{aligned}
$$

$$
\begin{aligned}
z_2 &\to -ik_\lambda + \xi_3 \\
\mathrm{Res}\{h^{(-)}(z_2)\} &= \frac{1}{\xi_3 - \xi_1} \, \frac{1 + N_\mathbf{p} - f_2}{\xi_3 - \xi_2 - \omega_\mathbf{p}} \left(\frac{1}{-ik_\lambda + \xi_3 + \omega_\mathbf{q}} - \frac{1}{-ik_\lambda + \xi_3 - \omega_\mathbf{q}} \right) \\
n_B(z_2) &= -f_3
\end{aligned}
$$

$$
\begin{aligned}
z_3 &\to \omega_\mathbf{q} \\
\mathrm{Res}\{h^{(-)}(z_3)\} &= \frac{1}{ik_\lambda + \omega_\mathbf{q} - \xi_1} \, \frac{1}{ik_\lambda + \omega_\mathbf{q} - \xi_3} \, (-1) \, \frac{1 + N_\mathbf{p} - f_2}{ik_\lambda + \omega_\mathbf{q} - \xi_2 - \omega_\mathbf{p}} \\
n_B(z_3) &= N_\mathbf{q}
\end{aligned}
$$

$$
z_4 \to -\omega_\mathbf{q}
$$

$$\begin{aligned}
\mathrm{Res}\{h^{(-)}(z_4)\} &= \frac{1}{ik_\lambda-\omega_{\mathbf{q}}-\xi_1}\ \frac{1}{ik_\lambda-\omega_{\mathbf{q}}-\xi_3}\ \frac{1+N_{\mathbf{p}}-f_2}{ik_\lambda-\omega_{\mathbf{q}}-\xi_2-\omega_{\mathbf{p}}}\\
n_B(z_4) &= -N_{\mathbf{q}}-1
\end{aligned}$$

$$\begin{aligned}
z_5 &\to -ik_\lambda+\xi_2+\omega_{\mathbf{p}}\\
\mathrm{Res}\{h^{(-)}(z_5)\} &= \frac{1}{\xi_2+\omega_{\mathbf{p}}-\xi_1}\ \frac{1}{\xi_2+\omega_{\mathbf{p}}-\xi_3}\left(\frac{1}{-ik_\lambda+\xi_2+\omega_{\mathbf{p}}+\omega_{\mathbf{q}}}-\frac{1}{-ik_\lambda+\xi_2+\omega_{\mathbf{p}}-\omega_{\mathbf{q}}}\right)(1+N_{\mathbf{p}}-f_2)\\
n_B(z_5) &= -\frac{N_{\mathbf{p}}f_2}{1+N_{\mathbf{p}}-f_2}
\end{aligned}$$

The terms related to the two-phonon processes are those containing $1/(ik-\xi_2\pm\omega_{\mathbf{p}}\pm\omega_{\mathbf{q}})$. After repeating this procedure for $h^{(+)}$, collecting terms and ignoring terms that are irrelevant to the two-phonon processes, we get

$$\begin{aligned}
\frac{1}{\beta^2}\sum_{i\omega_\kappa}\sum_{i\omega_\sigma} f(i\omega_\kappa,i\omega_\sigma) &= \frac{1}{ik_\lambda-\xi_2-\omega_{\mathbf{p}}+\omega_{\mathbf{q}}}\left[\frac{1+N_{\mathbf{p}}-f_2}{ik_\lambda-\xi_1+\omega_{\mathbf{q}}}\ \frac{N_{\mathbf{q}}}{ik_\lambda-\xi_3+\omega_{\mathbf{q}}}+\frac{N_{\mathbf{p}}f_2}{\xi_2-\xi_1+\omega_{\mathbf{p}}}\ \frac{1}{\xi_2-\xi_3+\omega_{\mathbf{p}}}\right]\\
&+\frac{1}{ik_\lambda-\xi_2-\omega_{\mathbf{p}}-\omega_{\mathbf{q}}}\left[\frac{1+N_{\mathbf{p}}-f_2}{ik_\lambda-\xi_1-\omega_{\mathbf{q}}}\ \frac{1+N_{\mathbf{q}}}{ik_\lambda-\xi_3-\omega_{\mathbf{q}}}-\frac{N_{\mathbf{p}}f_2}{\xi_2-\xi_1+\omega_{\mathbf{p}}}\ \frac{1}{\xi_2-\xi_3+\omega_{\mathbf{p}}}\right]\\
&+\frac{1}{ik_\lambda-\xi_2+\omega_{\mathbf{p}}+\omega_{\mathbf{q}}}\left[\frac{N_{\mathbf{p}}+f_2}{ik_\lambda-\xi_1+\omega_{\mathbf{q}}}\ \frac{N_{\mathbf{q}}}{ik_\lambda-\xi_3+\omega_{\mathbf{q}}}+\frac{f_2\,(1+N_{\mathbf{p}})}{\xi_2-\xi_1-\omega_{\mathbf{p}}}\ \frac{1}{\xi_2-\xi_3-\omega_{\mathbf{p}}}\right]\\
&+\frac{1}{ik_\lambda-\xi_2+\omega_{\mathbf{p}}-\omega_{\mathbf{q}}}\left[\frac{N_{\mathbf{p}}+f_2}{ik_\lambda-\xi_1-\omega_{\mathbf{q}}}\ \frac{1+N_{\mathbf{q}}}{ik_\lambda-\xi_3-\omega_{\mathbf{q}}}-\frac{f_2\,(1+N_{\mathbf{p}})}{\xi_2-\xi_1-\omega_{\mathbf{p}}}\ \frac{1}{\xi_2-\xi_3-\omega_{\mathbf{p}}}\right]\\
&+\cdots
\end{aligned}$$

The rates of the two-phonon processes emerge after analytically continuing ik_λ to $E+i\eta$ and taking the imaginary part of $1/(E-\xi_2\pm\omega_{\mathbf{p}}\pm\omega_{\mathbf{q}}+i\eta)$. We also use the delta functions to set $E=\xi_2\mp\omega_{\mathbf{p}}\mp\omega_{\mathbf{q}}$ in some of the denominators. After carrying out these calculations, we obtain

$$\begin{aligned}
\mathrm{Im}\left\{\frac{1}{\beta^2}\sum_{i\omega_\kappa}\sum_{i\omega_\sigma} f(i\omega_\kappa,i\omega_\sigma)\right\} &= -i\pi\delta(E-\xi_2-\omega_{\mathbf{p}}+\omega_{\mathbf{q}})\frac{1}{\xi_2-\xi_1+\omega_{\mathbf{p}}}\ \frac{1}{\xi_2-\xi_3+\omega_{\mathbf{p}}}\left[\,(1+N_{\mathbf{p}}-f_2)N_{\mathbf{q}}+N_{\mathbf{p}}f_2\,\right]\\
&-i\pi\delta(E-\xi_2-\omega_{\mathbf{p}}-\omega_{\mathbf{q}})\frac{1}{\xi_2-\xi_1+\omega_{\mathbf{p}}}\ \frac{1}{\xi_2-\xi_3+\omega_{\mathbf{p}}}\left[\,(1+N_{\mathbf{p}}-f_2)(1+N_{\mathbf{q}})-N_{\mathbf{p}}f_2\,\right]\\
&-i\pi\delta(E-\xi_2+\omega_{\mathbf{p}}+\omega_{\mathbf{q}})\frac{1}{\xi_2-\xi_1-\omega_{\mathbf{p}}}\ \frac{1}{\xi_2-\xi_3-\omega_{\mathbf{p}}}\left[\,(N_{\mathbf{p}}+f_2)N_{\mathbf{q}}+f_2(1+N_{\mathbf{p}})\,\right]\\
&-i\pi\delta(E-\xi_2+\omega_{\mathbf{p}}-\omega_{\mathbf{q}})\frac{1}{\xi_2-\xi_1-\omega_{\mathbf{p}}}\ \frac{1}{\xi_2-\xi_3-\omega_{\mathbf{p}}}\left[\,(N_{\mathbf{p}}+f_2)(1+N_{\mathbf{q}})-f_2(1+N_{\mathbf{p}})\,\right]\\
&+\cdots
\end{aligned}$$

Case with $n_3 = n_1$

In this case, $h^{(-)}$ is defined as

$$h^{(-)}(z)=\left(\frac{1}{z+ik_\lambda-\xi_1}\right)^2\ \frac{-2\omega_{\mathbf{q}}}{z^2-\omega_{\mathbf{q}}^2}\ \frac{1+N_{\mathbf{p}}-f_2}{z+ik_\lambda-\xi_2-\omega_{\mathbf{p}}}.$$

The function $h(z)n_B(z)$ has a pole of order 2 at $z_1=-ik_\lambda+\xi_1$. By employing

$$\mathrm{Res}\{f,z_1\}=\frac{1}{(n-1)!}\lim_{z\to z_1}\frac{d^{n-1}}{dz^{n-1}}\{(z-z_1)^n f(z)\},$$

where n is the order of the pole, we get

$$\mathrm{Res}\{h^{(-)}n_B,z_1\}=\frac{4z_1\omega_{\mathbf{q}}}{(z_1^2-\omega_{\mathbf{q}}^2)^2}\ \frac{1+N_{\mathbf{p}}-f_2}{z_1+ik_\lambda-\xi_2-\omega_{\mathbf{p}}}\ n_B(z_1)+\frac{2\omega_{\mathbf{q}}}{z_1^2-\omega_{\mathbf{q}}^2}\ \frac{1+N_{\mathbf{p}}-f_2}{(z_1+ik_\lambda-\xi_2-\omega_{\mathbf{p}})^2}\ n_B(z_1)$$

$$+\ \frac{-2\omega_{\mathbf{q}}}{z_1^2-\omega_{\mathbf{q}}^2}\ \frac{1+N_{\mathbf{p}}-f_2}{z_1+ik_\lambda-\xi_2-\omega_{\mathbf{p}}}\ n'_B(z_1).$$

After substituting $z_1=-ik_\lambda+\xi_1$, $n_B(z_1)=-f_1$ and $n'_B(z_1)=\beta f_1(1-f_1)$, we get

$$\mathrm{Res}\{h^{(-)}n_B,z_1\}=\frac{f_1(1+N_{\mathbf{p}}-f_2)}{(\xi_1-\xi_2-\omega_{\mathbf{p}})^2}\ \frac{-2\omega_{\mathbf{q}}}{(ik_\lambda-\xi_1)^2-\omega_{\mathbf{q}}^2}\ +\ \frac{\beta f_1(1-f_1)(1+N_{\mathbf{p}}-f_2)}{\xi_1-\xi_2-\omega_{\mathbf{p}}}\ \frac{-2\omega_{\mathbf{q}}}{(ik_\lambda-\xi_1)^2-\omega_{\mathbf{q}}^2}$$

$$-\ \frac{f_1(1+N_{\mathbf{p}}-f_2)}{\xi_1-\xi_2-\omega_{\mathbf{p}}}\left[\frac{1}{(ik_\lambda-\xi_1+\omega_{\mathbf{q}})^2}-\frac{1}{(ik_\lambda-\xi_1-\omega_{\mathbf{q}})^2}\right].$$

The other three poles are simple poles and can be treated in the usual way. Repeating this procedure for $h^{(+)}$ and adding all the contributions, we get

$$\frac{1}{\beta^2}\sum_{i\omega_\kappa}\sum_{i\omega_\sigma}f(i\omega_\kappa,i\omega_\sigma)=$$

$$\frac{2\omega_{\mathbf{q}}(N_{\mathbf{p}}+f_2)}{(ik_\lambda-\xi_1)^2-\omega_{\mathbf{q}}^2}\left[\frac{f_1}{(\xi_1-\xi_2+\omega_{\mathbf{p}})^2}+\frac{\beta f_1(1-f_1)}{\xi_1-\xi_2+\omega_{\mathbf{p}}}\right]+\ \frac{2\omega_{\mathbf{q}}(1+N_{\mathbf{p}}-f_2)}{(ik_\lambda-\xi_1)^2-\omega_{\mathbf{q}}^2}\left[\frac{f_1}{(\xi_1-\xi_2-\omega_{\mathbf{p}})^2}+\frac{\beta f_1(1-f_1)}{\xi_1-\xi_2-\omega_{\mathbf{p}}}\right]$$

$$-\ \frac{2\omega_{\mathbf{q}}f_2(1+N_{\mathbf{p}})}{(ik_\lambda-\xi_2+\omega_{\mathbf{p}})^2-\omega_{\mathbf{q}}^2}\left(\frac{1}{\xi_1-\xi_2+\omega_{\mathbf{p}}}\right)^2-\ \frac{2\omega_{\mathbf{q}}f_2N_{\mathbf{p}}}{(ik_\lambda-\xi_2-\omega_{\mathbf{p}})^2-\omega_{\mathbf{q}}^2}\left(\frac{1}{\xi_1-\xi_2-\omega_{\mathbf{p}}}\right)^2$$

$$+\ (N_{\mathbf{p}}+f_2)\left(\frac{1}{ik_\lambda-\xi_1+\omega_{\mathbf{q}}}\right)^2\ \left[\frac{f_1}{\xi_1-\xi_2+\omega_{\mathbf{p}}}+\frac{N_{\mathbf{q}}}{ik_\lambda-\xi_2+\omega_{\mathbf{p}}+\omega_{\mathbf{q}}}\right]$$

$$+\ (1+N_{\mathbf{p}}-f_2)\left(\frac{1}{ik_\lambda-\xi_1+\omega_{\mathbf{q}}}\right)^2\ \left[\frac{f_1}{\xi_1-\xi_2-\omega_{\mathbf{p}}}+\frac{N_{\mathbf{q}}}{ik_\lambda-\xi_2-\omega_{\mathbf{p}}+\omega_{\mathbf{q}}}\right]$$

$$+\ (N_{\mathbf{p}}+f_2)\left(\frac{1}{ik_\lambda-\xi_1-\omega_{\mathbf{q}}}\right)^2\ \left[\frac{-f_1}{\xi_1-\xi_2+\omega_{\mathbf{p}}}+\frac{1+N_{\mathbf{q}}}{ik_\lambda-\xi_2+\omega_{\mathbf{p}}-\omega_{\mathbf{q}}}\right]$$

$$+\ (1+N_{\mathbf{p}}-f_2)\left(\frac{1}{ik_\lambda-\xi_1-\omega_{\mathbf{q}}}\right)^2\ \left[\frac{-f_1}{\xi_1-\xi_2-\omega_{\mathbf{p}}}+\frac{1+N_{\mathbf{q}}}{ik_\lambda-\xi_2-\omega_{\mathbf{p}}-\omega_{\mathbf{q}}}\right]$$

$$=\ \frac{1}{ik_\lambda-\xi_2-\omega_{\mathbf{p}}+\omega_{\mathbf{q}}}\left[\ \frac{N_{\mathbf{q}}(1+N_{\mathbf{p}}-f_2)}{(ik_\lambda-\xi_1+\omega_{\mathbf{q}})^2}+\frac{N_{\mathbf{p}}f_2}{(\xi_2-\xi_1+\omega_{\mathbf{p}})^2}\ \right]$$

$$+\ \frac{1}{ik_\lambda-\xi_2-\omega_{\mathbf{p}}-\omega_{\mathbf{q}}}\left[\ \frac{(1+N_{\mathbf{q}})(1+N_{\mathbf{p}}-f_2)}{(ik_\lambda-\xi_1-\omega_{\mathbf{q}})^2}-\frac{N_{\mathbf{p}}f_2}{(\xi_2-\xi_1+\omega_{\mathbf{p}})^2}\ \right]$$

$$+\ \frac{1}{ik_\lambda-\xi_2+\omega_{\mathbf{p}}+\omega_{\mathbf{q}}}\left[\ \frac{N_{\mathbf{q}}(N_{\mathbf{p}}+f_2)}{(ik_\lambda-\xi_1+\omega_{\mathbf{q}})^2}+\frac{f_2\ (1+N_{\mathbf{p}})}{(\xi_2-\xi_1-\omega_{\mathbf{p}})^2}\ \right]$$

$$+\ \frac{1}{ik_\lambda-\xi_2+\omega_{\mathbf{p}}-\omega_{\mathbf{q}}}\left[\ \frac{(1+N_{\mathbf{q}})(N_{\mathbf{p}}+f_2)}{(ik_\lambda-\xi_1-\omega_{\mathbf{q}})^2}-\frac{f_2\ (1+N_{\mathbf{p}})}{(\xi_2-\xi_1-\omega_{\mathbf{p}})^2}\ \right]$$

$$+\cdots$$

We perform the analytic continuation $ik_\lambda\to E+i\eta$, take the imaginary part of $1/(E-\xi_2\pm\omega_{\mathbf{p}}\pm\omega_{\mathbf{q}}+i\eta)$, and get

$$\mathrm{Im}\left\{\frac{1}{\beta^2}\sum_{i\omega_\kappa}\sum_{i\omega_\sigma}f(i\omega_\kappa,i\omega_\sigma)\right\}\ =\ -\,i\pi\delta(E-\xi_2-\omega_{\mathbf{p}}+\omega_{\mathbf{q}})\frac{1}{(\xi_2-\xi_1+\omega_{\mathbf{p}})^2}\,[\,(1+N_{\mathbf{p}}-f_2)N_{\mathbf{q}}+N_{\mathbf{p}}f_2\,]$$

$$
\begin{aligned}
&- i\pi\delta(E-\xi_2-\omega_{\mathbf{p}}-\omega_{\mathbf{q}})\frac{1}{(\xi_2-\xi_1+\omega_{\mathbf{p}})^2}\,[\,(1+N_{\mathbf{p}}-f_2)(1+N_{\mathbf{q}})-N_{\mathbf{p}}f_2\,]\\
&- i\pi\delta(E-\xi_2+\omega_{\mathbf{p}}+\omega_{\mathbf{q}})\frac{1}{(\xi_2-\xi_1-\omega_{\mathbf{p}})^2}\,[\,(N_{\mathbf{p}}+f_2)N_{\mathbf{q}}+f_2(1+N_{\mathbf{p}})\,]\\
&- i\pi\delta(E-\xi_2+\omega_{\mathbf{p}}-\omega_{\mathbf{q}})\frac{1}{(\xi_2-\xi_1-\omega_{\mathbf{p}})^2}\,[\,(N_{\mathbf{p}}+f_2)(1+N_{\mathbf{q}})-f_2(1+N_{\mathbf{p}})\,]\\
&+\cdots
\end{aligned}
$$

Two-Loop Diagram IIb

The second two-loop diagram, called here IIb, is shown in the figure below. Since this diagram also has $L=2$ and $F=0$, the Feynman rules give

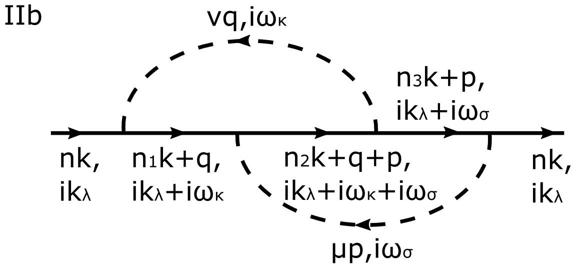

$$
\Sigma^{(\mathrm{IIb})}=\frac{1}{\beta^2N_\Omega^2}\sum_{n_1n_2n_3}\sum_{\nu\mathbf{q}}\sum_{\mu\mathbf{p}}g_{n_1n\nu}(\mathbf{k},\mathbf{q})g^*_{n_2n_3\nu}(\mathbf{k}+\mathbf{p},\mathbf{q})g_{n_2n_1\mu}(\mathbf{k}+\mathbf{q},\mathbf{p})g^*_{n_3n\mu}(\mathbf{k},\mathbf{p})\sum_{i\omega_\kappa}\sum_{i\omega_\sigma}f(i\omega_\kappa,i\omega_\sigma),
$$

where $f(i\omega_\kappa,i\omega_\sigma)$ is defined as

$$
\begin{aligned}
f(i\omega_\kappa,i\omega_\sigma)&\equiv\frac{1}{ik_\lambda+i\omega_\kappa-\xi_1}\;\frac{1}{ik_\lambda+i\omega_\kappa+i\omega_\sigma-\xi_2}\;\frac{1}{ik_\lambda+i\omega_\sigma-\xi_{3\mathbf{p}}}\;\frac{2\omega_{\mathbf{q}}}{-\omega_\kappa^2-\omega_{\mathbf{q}}^2}\;\frac{2\omega_{\mathbf{p}}}{-\omega_\sigma^2-\omega_{\mathbf{p}}^2}\\
&\equiv A_\kappa\frac{1}{ik_\lambda+i\omega_\kappa+i\omega_\sigma-\xi_2}\;\frac{1}{ik_\lambda+i\omega_\sigma-\xi_{3\mathbf{p}}}\;\frac{2\omega_{\mathbf{p}}}{-\omega_\sigma^2-\omega_{\mathbf{p}}^2}.
\end{aligned}
$$

Using a notation we introduced above, A_κ collects all terms independent of ω_σ, and we use again abbreviations introduced in the previous section, such as $\xi_{3\mathbf{p}}\equiv\xi_{n_3\mathbf{k}+\mathbf{p}}$, etc. Summing over $i\omega_\sigma$ first, we get

$$
\begin{aligned}
-\frac{1}{\beta}\sum_{i\omega_\sigma}f(i\omega_\kappa,i\omega_\sigma)=\;&f_2A_\kappa\frac{1}{i\omega_\kappa-\xi_2+\xi_{3\mathbf{p}}}\left(\frac{1}{ik_\lambda+i\omega_\kappa-\xi_2-\omega_{\mathbf{p}}}-\frac{1}{ik_\lambda+i\omega_\kappa-\xi_2+\omega_{\mathbf{p}}}\right)\\
&+f_{3\mathbf{p}}A_\kappa\frac{1}{i\omega_\kappa-\xi_2+\xi_{3\mathbf{p}}}\left(\frac{1}{ik_\lambda-\xi_{3\mathbf{p}}+\omega_{\mathbf{p}}}-\frac{1}{ik_\lambda-\xi_{3\mathbf{p}}-\omega_{\mathbf{p}}}\right)\\
&+N_{\mathbf{p}}A_\kappa\frac{1}{ik_\lambda+i\omega_\kappa-\xi_2+\omega_{\mathbf{p}}}\;\frac{1}{ik_\lambda-\xi_{3\mathbf{p}}+\omega_{\mathbf{p}}}+(N_{\mathbf{p}}+1)A_\kappa\frac{1}{ik_\lambda+i\omega_\kappa-\xi_2-\omega_{\mathbf{p}}}\;\frac{1}{ik_\lambda-\xi_{3\mathbf{p}}-\omega_{\mathbf{p}}}.
\end{aligned}
$$

We then sum over $i\omega_\kappa$, and collect the relevant terms for two-phonon scattering processes, which are given below:

$$
\begin{aligned}
&\frac{1}{\beta^2}\sum_{i\omega_\kappa}\sum_{i\omega_\sigma}f(i\omega_\kappa,i\omega_\sigma)\;=\\
&\quad\frac{1}{ik_\lambda-\xi_2-\omega_{\mathbf{p}}+\omega_{\mathbf{q}}}\Bigg[\frac{-f_2}{\xi_2-\xi_1+\omega_{\mathbf{p}}}\;\frac{1}{ik_\lambda-\xi_{3\mathbf{p}}-\omega_{\mathbf{p}}}\;\frac{f_2N_{\mathbf{p}}}{1-f_2+N_{\mathbf{p}}}+\frac{f_2}{ik_\lambda-\xi_1+\omega_{\mathbf{q}}}\;\frac{N_{\mathbf{q}}}{\xi_{3\mathbf{p}}-\xi_2+\omega_{\mathbf{q}}}\\
&\qquad+\frac{1+N_{\mathbf{p}}}{\xi_2-\xi_1+\omega_{\mathbf{p}}}\;\frac{1}{ik_\lambda-\xi_{3\mathbf{p}}-\omega_{\mathbf{p}}}\;\frac{f_2N_{\mathbf{p}}}{1-f_2+N_{\mathbf{p}}}+\frac{1+N_{\mathbf{p}}}{ik_\lambda-\xi_1+\omega_{\mathbf{q}}}\;\frac{N_{\mathbf{q}}}{ik_\lambda-\xi_{3\mathbf{p}}-\omega_{\mathbf{p}}}\Bigg]
\end{aligned}
$$

$$+\frac{1}{ik_\lambda-\xi_2-\omega_{\mathbf{p}}-\omega_{\mathbf{q}}}\left[\frac{f_2}{\xi_2-\xi_1+\omega_{\mathbf{p}}}\frac{1}{ik_\lambda-\xi_{3\mathbf{p}}-\omega_{\mathbf{p}}}\frac{f_2N_{\mathbf{p}}}{1-f_2+N_{\mathbf{p}}}+\frac{f_2}{ik_\lambda-\xi_1-\omega_{\mathbf{q}}}\frac{1+N_{\mathbf{q}}}{\xi_{3\mathbf{p}}-\xi_2-\omega_{\mathbf{q}}}\right.$$

$$\left.-\frac{1+N_{\mathbf{p}}}{\xi_2-\xi_1+\omega_{\mathbf{p}}}\frac{1}{ik_\lambda-\xi_{3\mathbf{p}}-\omega_{\mathbf{p}}}\frac{f_2N_{\mathbf{p}}}{1-f_2+N_{\mathbf{p}}}+\frac{1+N_{\mathbf{p}}}{ik_\lambda-\xi_1-\omega_{\mathbf{q}}}\frac{1+N_{\mathbf{q}}}{ik_\lambda-\xi_{3\mathbf{p}}-\omega_{\mathbf{p}}}\right]$$

$$+\frac{1}{ik_\lambda-\xi_2+\omega_{\mathbf{p}}+\omega_{\mathbf{q}}}\left[\frac{f_2}{\xi_2-\xi_1-\omega_{\mathbf{p}}}\frac{1}{ik_\lambda-\xi_{3\mathbf{p}}+\omega_{\mathbf{p}}}\frac{f_2(1+N_{\mathbf{p}})}{f_2+N_{\mathbf{p}}}-\frac{f_2}{ik_\lambda-\xi_1+\omega_{\mathbf{q}}}\frac{N_{\mathbf{q}}}{\xi_{3\mathbf{p}}-\xi_2+\omega_{\mathbf{q}}}\right.$$

$$\left.+\frac{N_{\mathbf{p}}}{\xi_2-\xi_1-\omega_{\mathbf{p}}}\frac{1}{ik_\lambda-\xi_{3\mathbf{p}}+\omega_{\mathbf{p}}}\frac{f_2(1+N_{\mathbf{p}})}{f_2+N_{\mathbf{p}}}+\frac{N_{\mathbf{p}}}{ik_\lambda-\xi_1+\omega_{\mathbf{q}}}\frac{N_{\mathbf{q}}}{ik_\lambda-\xi_{3\mathbf{p}}+\omega_{\mathbf{p}}}\right]$$

$$+\frac{1}{ik_\lambda-\xi_2+\omega_{\mathbf{p}}-\omega_{\mathbf{q}}}\left[\frac{-f_2}{\xi_2-\xi_1-\omega_{\mathbf{p}}}\frac{1}{ik_\lambda-\xi_{3\mathbf{p}}+\omega_{\mathbf{p}}}\frac{f_2(1+N_{\mathbf{p}})}{f_2+N_{\mathbf{p}}}-\frac{f_2}{ik_\lambda-\xi_1-\omega_{\mathbf{q}}}\frac{1+N_{\mathbf{q}}}{\xi_{3\mathbf{p}}-\xi_2-\omega_{\mathbf{q}}}\right.$$

$$\left.+\frac{-N_{\mathbf{p}}}{\xi_2-\xi_1-\omega_{\mathbf{p}}}\frac{1}{ik_\lambda-\xi_{3\mathbf{p}}+\omega_{\mathbf{p}}}\frac{f_2(1+N_{\mathbf{p}})}{f_2+N_{\mathbf{p}}}+\frac{N_{\mathbf{p}}}{ik_\lambda-\xi_1-\omega_{\mathbf{q}}}\frac{1+N_{\mathbf{q}}}{ik_\lambda-\xi_{3\mathbf{p}}+\omega_{\mathbf{p}}}\right]$$

$$+\cdots$$

After performing the analytic continuation and taking the imaginary part, we get

$$\begin{aligned}
&\mathrm{Im}\left\{\frac{1}{\beta^2}\sum_{i\omega_\kappa}\sum_{i\omega_\sigma}f(i\omega_\kappa,i\omega_\sigma)\right\}=\\
&-i\pi\delta(E-\xi_2-\omega_{\mathbf{p}}+\omega_{\mathbf{q}})\frac{1}{\xi_2-\xi_1+\omega_{\mathbf{p}}}\frac{1}{\xi_2-\xi_{3\mathbf{p}}-\omega_{\mathbf{q}}}\left[\,N_{\mathbf{q}}+N_{\mathbf{q}}N_{\mathbf{p}}+N_{\mathbf{p}}f_2-N_{\mathbf{q}}f_2\,\right]\\
&-i\pi\delta(E-\xi_2-\omega_{\mathbf{p}}-\omega_{\mathbf{q}})\frac{1}{\xi_2-\xi_1+\omega_{\mathbf{p}}}\frac{1}{\xi_2-\xi_{3\mathbf{p}}+\omega_{\mathbf{q}}}\left[\,(1+N_{\mathbf{p}})(1+N_{\mathbf{q}})-f_2(1+N_{\mathbf{p}}+N_{\mathbf{q}})\,\right]\\
&-i\pi\delta(E-\xi_2+\omega_{\mathbf{p}}+\omega_{\mathbf{q}})\frac{1}{\xi_2-\xi_1-\omega_{\mathbf{p}}}\frac{1}{\xi_2-\xi_{3\mathbf{p}}-\omega_{\mathbf{q}}}\left[\,N_{\mathbf{p}}N_{\mathbf{q}}+N_{\mathbf{q}}f_2+N_{\mathbf{p}}f_2+f_2\,\right]\\
&-i\pi\delta(E-\xi_2+\omega_{\mathbf{p}}-\omega_{\mathbf{q}})\frac{1}{\xi_2-\xi_1-\omega_{\mathbf{p}}}\frac{1}{\xi_2-\xi_{3\mathbf{p}}+\omega_{\mathbf{q}}}\left[\,N_{\mathbf{p}}+N_{\mathbf{q}}N_{\mathbf{p}}+N_{\mathbf{q}}f_2-N_{\mathbf{p}}f_2\,\right]\\
&+\cdots
\end{aligned}$$

Two-Phonon Scattering Rates

Collecting the contributions from diagrams IIa and IIb, using $\Gamma=-(2/\hbar)\mathrm{Im}\Sigma$, and setting E to the band energy $\xi_{n\mathbf{k}}$, the scattering rate of the two-phonon processes becomes

$$\Gamma^{(2\mathrm{ph})}_{n\mathbf{k}}=\frac{2\pi}{\hbar}\frac{1}{N_\Omega^2}\sum_{n_1n_2n_3}\sum_{\mathbf{qp}}\sum_{\nu\mu}\Big[\gamma^{(\mathrm{i})}\delta(\xi_{n\mathbf{k}}-\xi_2-\omega_{\mathbf{p}}+\omega_{\mathbf{q}})+\gamma^{(\mathrm{ii})}\delta(\xi_{n\mathbf{k}}-\xi_2-\omega_{\mathbf{p}}-\omega_{\mathbf{q}})$$
$$+\gamma^{(\mathrm{iii})}\delta(\xi_{n\mathbf{k}}-\xi_2+\omega_{\mathbf{p}}+\omega_{\mathbf{q}})+\gamma^{(\mathrm{iv})}\delta(\xi_{n\mathbf{k}}-\xi_2+\omega_{\mathbf{p}}-\omega_{\mathbf{q}})\Big]\,,$$

where we introduce the process amplitudes

$$\gamma^{(\mathrm{i})}=(N_{\mathbf{q}}+N_{\mathbf{q}}N_{\mathbf{p}}+N_{\mathbf{p}}f_2-N_{\mathbf{q}}f_2)\times$$

$$\frac{g_{n_1n\nu}(\mathbf{k},\mathbf{q})g_{n_2n_1\mu}(\mathbf{k}+\mathbf{q},\mathbf{p})}{\xi_2-\xi_1+\omega_{\mathbf{p}}}\left(\frac{g^*_{n_3n\nu}(\mathbf{k},\mathbf{q})g^*_{n_2n_3\mu}(\mathbf{k}+\mathbf{q},\mathbf{p})}{\xi_2-\xi_3+\omega_{\mathbf{p}}}+\frac{g^*_{n_2n_3\nu}(\mathbf{k}+\mathbf{p},\mathbf{q})g^*_{n_3n\mu}(\mathbf{k},\mathbf{p})}{\xi_2-\xi_{3\mathbf{p}}-\omega_{\mathbf{q}}}\right)$$

$$\gamma^{(\mathrm{ii})}=[(1+N_{\mathbf{q}})(1+N_{\mathbf{p}}-f_2)-N_{\mathbf{p}}f_2]\times$$

$$\frac{g_{n_1n\nu}(\mathbf{k},\mathbf{q})g_{n_2n_1\mu}(\mathbf{k}+\mathbf{q},\mathbf{p})}{\xi_2-\xi_1+\omega_{\mathbf{p}}}\left(\frac{g^*_{n_3n\nu}(\mathbf{k},\mathbf{q})g^*_{n_2n_3\mu}(\mathbf{k}+\mathbf{q},\mathbf{p})}{\xi_2-\xi_3+\omega_{\mathbf{p}}}+\frac{g^*_{n_2n_3\nu}(\mathbf{k}+\mathbf{p},\mathbf{q})g^*_{n_3n\mu}(\mathbf{k},\mathbf{p})}{\xi_2-\xi_{3\mathbf{p}}+\omega_{\mathbf{q}}}\right)$$

$$\begin{aligned}\gamma^{(\mathrm{iii})} &= [N_{\mathbf{q}}(N_{\mathbf{p}}+f_2)+(1+N_{\mathbf{p}})f_2]\times \\ &\quad \frac{g_{n_1n\nu}(\mathbf{k},\mathbf{q})g_{n_2n_1\mu}(\mathbf{k}+\mathbf{q},\mathbf{p})}{\xi_2-\xi_1-\omega_{\mathbf{p}}}\left(\frac{g^*_{n_3n\nu}(\mathbf{k},\mathbf{q})g^*_{n_2n_3\mu}(\mathbf{k}+\mathbf{q},\mathbf{p})}{\xi_2-\xi_3-\omega_{\mathbf{p}}}+\frac{g^*_{n_2n_3\nu}(\mathbf{k}+\mathbf{p},\mathbf{q})g^*_{n_3n\mu}(\mathbf{k},\mathbf{p})}{\xi_2-\xi_{3\mathbf{p}}-\omega_{\mathbf{q}}}\right)\end{aligned}$$

$$\begin{aligned}\gamma^{(\mathrm{iv})} &= (N_{\mathbf{p}}+N_{\mathbf{q}}N_{\mathbf{p}}+N_{\mathbf{q}}f_2-N_{\mathbf{p}}f_2)\times \\ &\quad \frac{g_{n_1n\nu}(\mathbf{k},\mathbf{q})g_{n_2n_1\mu}(\mathbf{k}+\mathbf{q},\mathbf{p})}{\xi_2-\xi_1-\omega_{\mathbf{p}}}\left(\frac{g^*_{n_3n\nu}(\mathbf{k},\mathbf{q})g^*_{n_2n_3\mu}(\mathbf{k}+\mathbf{q},\mathbf{p})}{\xi_2-\xi_3-\omega_{\mathbf{p}}}+\frac{g^*_{n_2n_3\nu}(\mathbf{k}+\mathbf{p},\mathbf{q})g^*_{n_3n\mu}(\mathbf{k},\mathbf{p})}{\xi_2-\xi_{3\mathbf{p}}+\omega_{\mathbf{q}}}\right).\end{aligned}$$

Now we restore the infinitesimal $i\eta$ for the intermediate propagators. A useful sanity check is that our finite temperature results should reduce to the zero temperature results in the $T \to 0$ limit, from which we check that the pole structure of the finite and zero temperature expressions are consistent with each other. At zero temperature, we can directly compute the scattering amplitude $\mathcal{M}$. The zero temperature Feynman rules {see Eqs. (2.124) to (2.127) in Ref. [2]} give

$$\mathcal{M} \sim \frac{gg}{E-\xi+i\eta}.$$

The scattering rate is proportional to the absolute square of the scattering amplitude:

$$\Gamma \sim |\mathcal{M}|^2 \sim \left|\frac{gg}{E-\xi+i\eta}\right|^2.$$

Therefore, we will insert the infinitesimals in a way that allows us to express the scattering rates in an absolute square form. To achieve this, first note that quantities such as $\mathbf{q}$, $\mathbf{p}$, ν and μ are dummy variables that are summed over or integrated, so we can rename them at will. Let us denote $(\nu\mathbf{q} \leftrightarrow \mu\mathbf{p})$ the term with its dummy variables swapped in the way indicated by the arrows, $\nu \leftrightarrow \mu$, $\mathbf{q} \leftrightarrow \mathbf{p}$, etc. Let us consider the processes with one phonon absorption and one phonon emission first, that is, the sum of terms (i) and (iv):

$$\begin{aligned}&\sum_{n_1n_3}\left[\gamma^{(\mathrm{i})}\delta(\xi_{n\mathbf{k}}-\xi_2-\omega_{\mu\mathbf{p}}+\omega_{\nu\mathbf{q}})+\gamma^{(\mathrm{iv})}\delta(\xi_{n\mathbf{k}}-\xi_2+\omega_{\mu\mathbf{p}}-\omega_{\nu\mathbf{q}})\right] \\ &\quad = \sum_{n_1n_3}\left[\gamma^{(\mathrm{i})}\delta(\xi_{n\mathbf{k}}-\xi_2-\omega_{\mu\mathbf{p}}+\omega_{\nu\mathbf{q}})+\gamma^{(\mathrm{iv})}(\nu\mathbf{q}\leftrightarrow\mu\mathbf{p})\delta(\xi_{n\mathbf{k}}-\xi_2+\omega_{\mu\mathbf{p}}-\omega_{\nu\mathbf{q}})(\nu\mathbf{q}\leftrightarrow\mu\mathbf{p})\right] \\ &\quad \equiv \gamma^{(1\mathrm{e}1\mathrm{a})}\,\delta(\xi_{n\mathbf{k}}-\xi_2-\omega_{\mu\mathbf{p}}+\omega_{\nu\mathbf{q}}),\end{aligned}$$

where

$$\begin{aligned}\gamma^{(1\mathrm{e}1\mathrm{a})} &= \sum_{n_1n_3}\left[\gamma^{(\mathrm{i})}+\gamma^{(\mathrm{iv})}(\nu\mathbf{q}\leftrightarrow\mu\mathbf{p})\right] \\ &= (N_{\mathbf{q}}+N_{\mathbf{q}}N_{\mathbf{p}}+N_{\mathbf{p}}f_2-N_{\mathbf{q}}f_2)\sum_{n_1}\left(\frac{g_{n_1n\nu}(\mathbf{k},\mathbf{q})g_{n_2n_1\mu}(\mathbf{k}+\mathbf{q},\mathbf{p})}{\xi_2-\xi_1+\omega_{\mathbf{p}}}+\frac{g_{n_1n\mu}(\mathbf{k},\mathbf{p})g_{n_2n_1\nu}(\mathbf{k}+\mathbf{p},\mathbf{q})}{\xi_2-\xi_{1\mathbf{p}}-\omega_{\mathbf{q}}}\right) \\ &\quad \times\sum_{n_3}\left(\frac{g^*_{n_3n\nu}(\mathbf{k},\mathbf{q})g^*_{n_2n_3\mu}(\mathbf{k}+\mathbf{q},\mathbf{p})}{\xi_2-\xi_3+\omega_{\mathbf{p}}}+\frac{g^*_{n_2n_3\nu}(\mathbf{k}+\mathbf{p},\mathbf{q})g^*_{n_3n\mu}(\mathbf{k},\mathbf{p})}{\xi_2-\xi_{3\mathbf{p}}-\omega_{\mathbf{q}}}\right) \\ &= (N_{\mathbf{q}}+N_{\mathbf{q}}N_{\mathbf{p}}+N_{\mathbf{p}}f_2-N_{\mathbf{q}}f_2)\left|\sum_{n_1}\left(\frac{g_{n_1n\nu}(\mathbf{k},\mathbf{q})g_{n_2n_1\mu}(\mathbf{k}+\mathbf{q},\mathbf{p})}{\xi_2-\xi_1+\omega_{\mathbf{p}}+i\eta}+\frac{g_{n_1n\mu}(\mathbf{k},\mathbf{p})g_{n_2n_1\nu}(\mathbf{k}+\mathbf{p},\mathbf{q})}{\xi_2-\xi_{1\mathbf{p}}-\omega_{\mathbf{q}}+i\eta}\right)\right|^2.\end{aligned}$$

Since the expression is already in square form, we have inserted the $i\eta$ terms in a way that makes the expression become an absolute square. Using a similar approach for the process in which the electron emits two phonons,

$$\begin{aligned}\gamma^{(2\mathrm{e})} &= \sum_{n_1 n_3} \gamma^{(\mathrm{ii})} \\ &= \sum_{n_1 n_3} \frac{1}{2}\left[\gamma^{(\mathrm{ii})} + \gamma^{(\mathrm{ii})}\right] \\ &= \sum_{n_1 n_3} \frac{1}{2}\left[\gamma^{(\mathrm{ii})} + \gamma^{(\mathrm{ii})}(\nu\mathbf{q} \leftrightarrow \mu\mathbf{p})\right] \\ &= \frac{1}{2}\left[(1+N_{\mathbf{q}})(1+N_{\mathbf{p}}-f_2) - N_{\mathbf{p}} f_2\right] \left|\sum_{n_1}\left(\frac{g_{n_1 n\nu}(\mathbf{k},\mathbf{q}) g_{n_2 n_1 \mu}(\mathbf{k}+\mathbf{q},\mathbf{p})}{\xi_2 - \xi_1 + \omega_{\mathbf{p}} + i\eta} + \frac{g_{n_1 n\mu}(\mathbf{k},\mathbf{p}) g_{n_2 n_1 \nu}(\mathbf{k}+\mathbf{p},\mathbf{q})}{\xi_2 - \xi_{1\mathbf{p}} + \omega_{\mathbf{q}} + i\eta}\right)\right|^2,\end{aligned}$$

and for the process in which the electron absorbs two phonons,

$$\begin{aligned}\gamma^{(2\mathrm{a})} &= \sum_{n_1 n_3} \gamma^{(\mathrm{iii})} \\ &= \sum_{n_1 n_3} \frac{1}{2}\left[\gamma^{(\mathrm{iii})} + \gamma^{(\mathrm{iii})}\right] \\ &= \sum_{n_1 n_3} \frac{1}{2}\left[\gamma^{(\mathrm{iii})} + \gamma^{(\mathrm{iii})}(\nu\mathbf{q} \leftrightarrow \mu\mathbf{p})\right] \\ &= \frac{1}{2}\left[N_{\mathbf{q}}(N_{\mathbf{p}}+f_2) + (1+N_{\mathbf{p}}) f_2\right] \left|\sum_{n_1}\left(\frac{g_{n_1 n\nu}(\mathbf{k},\mathbf{q}) g_{n_2 n_1 \mu}(\mathbf{k}+\mathbf{q},\mathbf{p})}{\xi_2 - \xi_1 - \omega_{\mathbf{p}} + i\eta} + \frac{g_{n_1 n\mu}(\mathbf{k},\mathbf{p}) g_{n_2 n_1 \nu}(\mathbf{k}+\mathbf{p},\mathbf{q})}{\xi_2 - \xi_{1\mathbf{p}} - \omega_{\mathbf{q}} + i\eta}\right)\right|^2.\end{aligned}$$

We thus get:

$$\Gamma^{(2\mathrm{ph})}_{n\mathbf{k}} = \frac{2\pi}{\hbar}\frac{1}{N_\Omega^2}\sum_{n_2}\sum_{\mathbf{q}\,\mathbf{p}}\sum_{\nu\mu}\left[\gamma^{(1\mathrm{e}1\mathrm{a})}\delta(\xi_{n\mathbf{k}} - \xi_2 - \omega_{\mathbf{p}} + \omega_{\mathbf{q}}) + \gamma^{(2\mathrm{e})}\delta(\xi_{n\mathbf{k}} - \xi_2 - \omega_{\mathbf{p}} - \omega_{\mathbf{q}}) + \gamma^{(2\mathrm{a})}\delta(\xi_{n\mathbf{k}} - \xi_2 + \omega_{\mathbf{p}} + \omega_{\mathbf{q}})\right].$$

Resonance

The last expression is very close to the final result given in Eqs. (1)−(4) of the main text. The last problem we need to solve is that the sum giving $\Gamma^{(2\mathrm{ph})}_{n\mathbf{k}}$ in the expression above diverges when the intermediate electron state is on shell, in which case the denominator in the γ terms given above vanishes, resulting in a divergent scattering rate. This phenomenon is called resonance. The problem is that the intermediate state will eventually transition into a different state, but using free propagators $1/(E - \xi + i\eta)$ for the on shell intermediate states implies an infinite intermediate state lifetime. The common practice in this situation, which also arises in other quantum field theories, is to consider the full electron propagator $1/(E - \xi + i\eta - \Sigma)$ as shown in the figure below, which introduces a finite lifetime for the intermediate electronic state. Diagramatically, this approach is equivalent to performing a resummation of diagrams to all orders, as is done in the well-known GW self-energy {see Eq. (5.54) in Ref. [2]}. For our 2ph scattering rate expression, we simply add the intermediate state self-energy in the denominators of all the γ terms above, which removes the divergences.

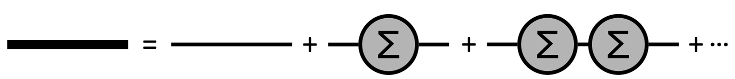

Summary

We rewrite the expression in more compact form. Defining the momentum of the final electronic state as $\mathbf{k}' \equiv \mathbf{k}+\mathbf{q}+\mathbf{p}$, and using the following constants

$$\alpha_{\mathbf{p}}^{(1e1a)} = \ 1,\ \ \alpha_{\mathbf{p}}^{(2e)} = 1,\ \ \alpha_{\mathbf{p}}^{(2a)} = -1,\ \ \alpha_{\mathbf{q}}^{(1e1a)} = -1,\ \ \alpha_{\mathbf{q}}^{(2e)} = 1,\ \ \alpha_{\mathbf{q}}^{(2a)} = -1,$$

we can write

$$\Gamma_{n\mathbf{k}}^{(2ph)} = \frac{2\pi}{\hbar}\frac{1}{N_\Omega^2}\sum_{n_2}\sum_{\nu\mathbf{q}}\sum_{\mu\mathbf{p}}\left[\widetilde{\Gamma}^{(1e1a)} + \widetilde{\Gamma}^{(2e)} + \widetilde{\Gamma}^{(2a)}\right],$$

where

$$\widetilde{\Gamma}^{(i)} = \gamma^{(i)}\ \delta(\xi_{n\mathbf{k}} - \xi_{n_2\mathbf{k}'} - \alpha_{\mathbf{p}}^{(i)}\omega_{\mu\mathbf{p}} - \alpha_{\mathbf{q}}^{(i)}\omega_{\nu\mathbf{q}}).$$

The square amplitudes $\gamma^{(i)}$ for the different processes, i = 1e1a, 2e and 2a, are defined as

$$\gamma^{(i)} = A^{(i)}\left|\sum_{n_1}\left(\frac{g_{n_1 n\nu}(\mathbf{k},\mathbf{q})g_{n_2 n_1\mu}(\mathbf{k}+\mathbf{q},\mathbf{p})}{\xi_{n_2\mathbf{k}'} - \xi_{n_1\mathbf{k}+\mathbf{q}} + \alpha_{\mathbf{p}}^{(i)}\omega_{\mu\mathbf{p}} + i\eta - \Sigma_{n_1\mathbf{k}+\mathbf{q}}} + \frac{g_{n_1 n\mu}(\mathbf{k},\mathbf{p})g_{n_2 n_1\nu}(\mathbf{k}+\mathbf{p},\mathbf{q})}{\xi_{n_2\mathbf{k}'} - \xi_{n_1\mathbf{k}+\mathbf{p}} + \alpha_{\mathbf{q}}^{(i)}\omega_{\nu\mathbf{q}} + i\eta - \Sigma_{n_1\mathbf{k}+\mathbf{p}}}\right)\right|^2, \quad (1)$$

where we have taken into account the resonance by adding the intermediate state self-energy in the denominators. The factors of $A^{(i)}$ contain the thermal occupation numbers of electrons and phonons, and are defined as

$$\begin{aligned} A^{(1e1a)} &= N_{\nu\mathbf{q}} + N_{\nu\mathbf{q}}N_{\mu\mathbf{p}} + N_{\mu\mathbf{p}}f_{n_2\mathbf{k}'} - N_{\nu\mathbf{q}}f_{n_2\mathbf{k}'}, \\ A^{(2e)} &= \frac{1}{2}\left[(1+N_{\nu\mathbf{q}})(1+N_{\mu\mathbf{p}} - f_{n_2\mathbf{k}'}) - N_{\mu\mathbf{p}}f_{n_2\mathbf{k}'}\right], \quad (2) \\ A^{(2a)} &= \frac{1}{2}\left[N_{\nu\mathbf{q}}(N_{\mu\mathbf{p}} + f_{n_2\mathbf{k}'}) + (1+N_{\mu\mathbf{p}})f_{n_2\mathbf{k}'}\right]. \end{aligned}$$

These are our final expressions for the two-phonon scattering rates, which are given in Eqs. (1)−(4) of the main text.

Supplementary Note 2: Temperature Dependence of the Two-Phonon Scattering Rates

Supplementary Figure 5 below shows the temperature dependence of the ratios of the 2ph scattering rate and the leading order scattering rate, $\Gamma^{(2ph)}/\Gamma^{(1ph)}$. Results are given for three electronic states, one in each of the regions I, II

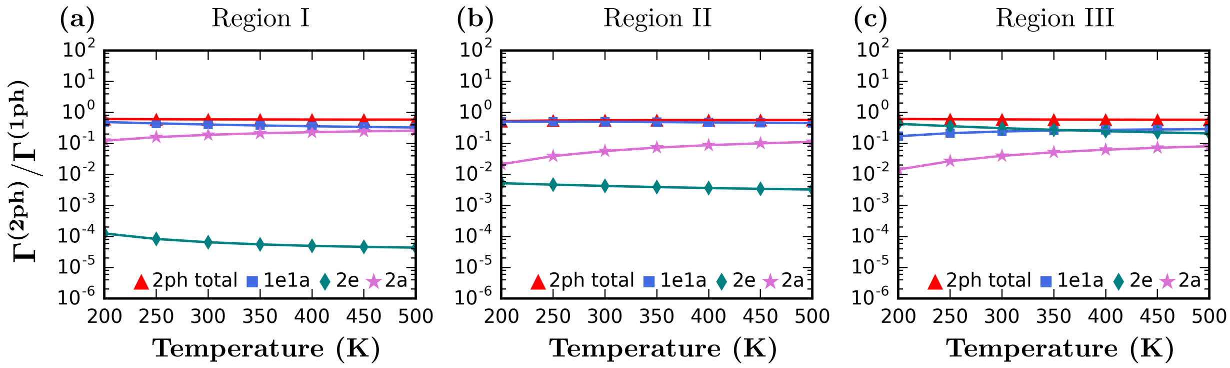

Supplementary Figure 5. **Temperature dependence of the 2ph scattering rates.** Temperature dependence of the ratios of the 2ph scattering processes to the leading order e-ph scattering rate. From left to right, the panels are for electronic states with energies of 20, 45 and 90 meV above the conduction band minimum, and thus respectively in region I, II and III defined in the main text.

and III defined in the main text. The ratios are give for both the total 2ph scattering rate and (for completeness) for the individual 2ph processes, 1e1a, 2e and 2a. In the 200−500 K temperature range considered in this work, the ratio of the total 2ph scattering rate to the 1ph scattering rate is nearly temperature independent in all three energy regions.

Supplementary Note 3: Boltzmann Transport Equation with Two-Phonon Contributions

Let us briefly summarize the formulation of the linearized Boltzmann transport equation (BTE) incorporating the 2ph scattering processes. A more extensive explanation of its formulation and derivation will be presented in a separate work. Defining the total e-ph scattering rate as

$$\Gamma_{n\mathbf{k}} = \Gamma^{(1\mathrm{ph})}_{n\mathbf{k}} + \Gamma^{(2\mathrm{ph})}_{n\mathbf{k}} \equiv \frac{1}{N_\Omega}\sum_m \sum_{\nu\mathbf{q}} \widetilde{\Gamma}^{(1\mathrm{ph})}_{n\mathbf{k},\,\nu\mathbf{q}} + \frac{1}{N_\Omega^2}\sum_{n_2}\sum_{\nu\mathbf{q}}\sum_{\mu\mathbf{p}} \widetilde{\Gamma}^{(2\mathrm{ph})}_{n\mathbf{k},\,\nu\mathbf{q},\,\mu\mathbf{p}}\ ,$$

the linearized BTE can be expressed as

$$\mathbf{F}_{n\mathbf{k}} = \mathbf{F}^0_{n\mathbf{k}} + \tau_{n\mathbf{k}}\left[\frac{1}{N_\Omega}\sum_m\sum_{\nu\mathbf{q}} \mathbf{F}_{m\mathbf{k}+\mathbf{q}} \widetilde{\Gamma}^{(1\mathrm{ph})}_{n\mathbf{k},\,\nu\mathbf{q}} + \frac{1}{N_\Omega^2}\sum_{n_2}\sum_{\nu\mathbf{q}}\sum_{\mu\mathbf{p}} \mathbf{F}_{n_2\mathbf{k}'} \widetilde{\Gamma}^{(2\mathrm{ph})}_{n\mathbf{k},\,\nu\mathbf{q},\,\mu\mathbf{p}}\right], \tag{3}$$

where the relaxation time $\tau_{n\mathbf{k}}$ is the inverse of the total scattering rate, namely $\tau_{n\mathbf{k}} = 1/\Gamma_{n\mathbf{k}}$, and the first and second terms in brackets are due to the lowest-order (1ph) and 2ph scattering processes, respectively. The function $\mathbf{F}_{n\mathbf{k}}$ is the unknown in the equation, and $\mathbf{F}^0_{n\mathbf{k}} = \tau_{n\mathbf{k}}\mathbf{v}_{n\mathbf{k}}$, with $\mathbf{v}_{n\mathbf{k}}$ the band velocity. After solving the equation, the electrical mobility in direction i can be obtained using

$$\mu^i = \frac{2e\beta}{n_c V_{\mathrm{uc}}}\sum_{n\mathbf{k}} f_{n\mathbf{k}}(1-f_{n\mathbf{k}})v^i_{n\mathbf{k}}F^i_{n\mathbf{k}},$$

where V_{uc} is the unit cell volume and n_c the charge carrier concentration.

A common approach to computing $\mathbf{F}_{n\mathbf{k}}$ is the relaxation time approximation (RTA), which neglects the second term on the right hand side of Eq. (3) and approximates $\mathbf{F}_{n\mathbf{k}}$ to $\mathbf{F}^0_{n\mathbf{k}}$. A more accurate solution can be obtained through an iterative approach (ITA) [3], in which, starting from the RTA solution $\mathbf{F}_{n\mathbf{k}} = \mathbf{F}^0_{n\mathbf{k}}$, one iteratively substitutes $\mathbf{F}_{n\mathbf{k}}$ in the right hand side of Eq. (3) to obtain an updated $\mathbf{F}_{n\mathbf{k}}$, until a converged $\mathbf{F}_{n\mathbf{k}}$ is reached.

Our work presents calculations, both within the RTA and ITA, in which the 2ph processes are either included or neglected; when only 1ph processes are included, $\tau_{n\mathbf{k}}$ is set to the inverse of $\Gamma^{(1\mathrm{ph})}_{n\mathbf{k}}$, and the second term in brackets in Eq. (3) is neglected. The ITA with 2ph contributions included is the most accurate level of theory, and the one that agrees best with experiment, while the ITA with only 1ph processes overestimates the experimental result. Note that interference between 2ph processes is neglected in the mobility calculation. This is a good approximation if for most of the ($\mathbf{q}$, $\mathbf{p}$) pairs there is only one process in Eq. (3) in the main text dominates, as in the case of GaAs that we have verified.

Supplementary References

[1] H. J. Kim, U. Kim, T. H. Kim, J. Kim, H. M. Kim, B.-G. Jeon, W.-J. Lee, H. S. Mun, K. T. Hong, J. Yu, K. Char, and K. H. Kim, Phys. Rev. B **86**, 165205 (2012).

[2] G. D. Mahan, *Many-Particle Physics*, 3rd ed. (Springer, 2000).

[3] W. Li, Phys. Rev. B **92**, 075405 (2015).
